# Machine learning-causal inference based on multi-omics data reveals the association of altered gut bacteria and bile acid metabolism with neonatal jaundice

**DOI:** 10.1080/19490976.2024.2388805

**Published:** 2024-08-21

**Authors:** Wanling Chen, Peng Zhang, Xueli Zhang, Tiantian Xiao, Jianhai Zeng, Kaiping Guo, Huixian Qiu, Guoqiang Cheng, Zhangxing Wang, Wenhao Zhou, Shujuan Zeng, Mingbang Wang

**Affiliations:** a Division of Neonatology, Longgang Central Hospital of Shenzhen, Shenzhen, China; b Shenzhen Clinical Medical College, Guangzhou University of Chinese Medicine, Shenzhen, China; c Division of Neonatology, Children’s Hospital of Fudan University, Shanghai, China; d Division of Neonatology, Shenzhen Longhua People’s Hospital, Shenzhen, China; e Department of Neonatology, Chengdu Women’s and Children’s Central Hospital, School of Medicine, University of Electronic Science and Technology of China, Chengdu, China; f Division of Pediatric, Longgang Central Hospital of Shenzhen, Shenzhen, China; g Division of Neonatology, Guangzhou Women and Children’s Medical Center, Guangzhou Medical University, Guangzhou, China; h Department of Neonatology, Longgang Maternity and Child Institute of Shantou University Medical College (Longgang District Maternity & Child Healthcare Hospital of Shenzhen City), Shenzhen, China; i Microbiome Therapy Center, Department of Experiment & Research, South China Hospital, Medical School, Shenzhen University, Shenzhen, China

**Keywords:** Neonatal jaundice, 16S rRNA gene sequencing, LC-MS/MS, Lasso, causal inference

## Abstract

Early identification of neonatal jaundice (NJ) appears to be essential to avoid bilirubin encephalopathy and neurological sequelae. The interaction between gut microbiota and metabolites plays an important role in early life. It is unclear whether the composition of the gut microbiota and metabolites can be used as an early indicator of NJ or to aid clinical decision-making. This study involved a total of 196 neonates and conducted two rounds of “discovery-validation” research on the gut microbiome-metabolome. It utilized methods of machine learning, causal inference, and clinical prediction model evaluation to assess the significance of gut microbiota and metabolites in classifying neonatal jaundice (NJ), as well as the potential causal relationships between corresponding clinical variables and NJ. In the discovery stage, NJ-associated gut microbiota, network modules, and metabolite composition were identified by gut microbiome-metabolome association analysis. The NJ-associated gut microbiota was closely related to bile acid metabolites. By Lasso machine learning assessment, we found that the gut bacteria were associated with abnormal bile acid metabolism. The machine learning-causal inference approach revealed that gut bacteria affected serum total bilirubin and NJ by influencing bile acid metabolism. NJ-associated gut bile acids are potential biomarkers of NJ, and clinical prediction models constructed based on these biomarkers have some clinical effects and the model may be used for disease risk prediction. In the validation stage, it was found that intestinal metabolites can predict NJ, and the machine learning-causal inference approach revealed that bile acid metabolites affected NJ itself by affecting the total bilirubin content. Intestinal bile acid metabolites are potential biomarkers of NJ. By applying machine learning-causal inference methods to gut microbiome-metabolome association studies, we found NJ-associated intestinal bacteria and their network modules and bile acid metabolite composition. The important role of intestinal bacteria and bile acid metabolites in NJ was determined, which can predict the risk of NJ.

## HIGHLIGHT

- Association analysis of the intestinal microbiome-metabolome found that neonatal jaundice (NJ)-related intestinal microbiota, network modules and metabolite composition, and the intestinal microbiota are closely related to bile acid metabolites.

- Gut bacteria were found to affect serum total bilirubin (TBIL) and NJ by influencing bile acid metabolism through a machine learning-causal inference approach, and bile acid metabolites affected NJ itself by affecting the TBIL content.

- NJ-associated gut bacteria and bile acids are potential biomarkers of NJ, and clinical decision-making models based on these biomarkers have some clinical effects for disease risk prediction.

## GRAPHICAL ABSTRACT

**Figure:**
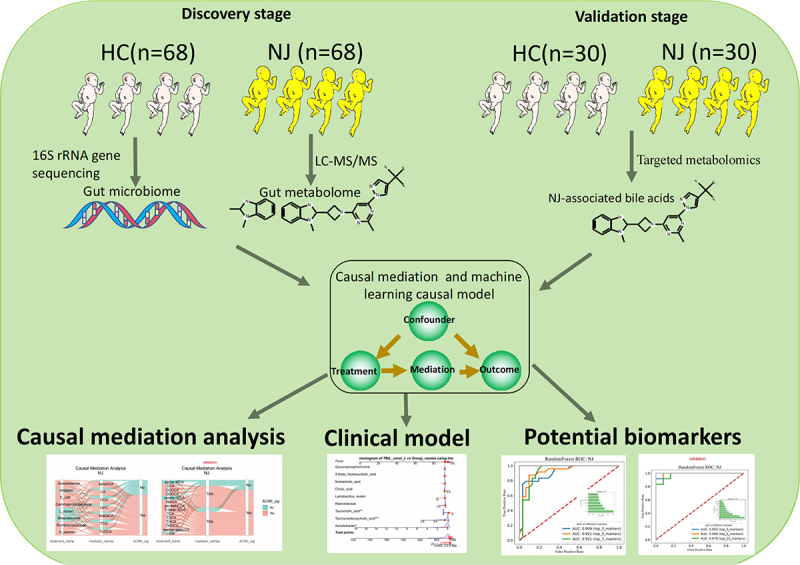

## Introduction

Neonatal jaundice (NJ), also known as neonatal hyperbilirubinemia, is clinically characterized by elevated total bilirubin (TBIL) levels in serum and is a common clinical presentation during the neonatal period. In most cases, jaundice resolves spontaneously. However, a small percentage of infants may develop severe hyperbilirubinemia or bilirubin encephalopathy, which can lead to brain damage or death if not diagnosed and treated promptly.^1,2^ Early identification of NJ is essential to avoid bilirubin encephalopathy.^3^ Causes of NJ are complicated. After birth, excess red blood cells are destroyed, leading to excessive bilirubin production. In addition, the metabolic function of the newborn is underdeveloped, resulting in slower and less efficient bilirubin metabolism. Finally, factors such as infection, hypoxia, hemorrhage, and gut dysbiosis can contribute to elevated TBIL levels,^1,4,5^

The gut microbiota plays an important role in human health, and disruptions in this microbiota during the newborn stage may have major implications on the development of the immune system and adult health.^6,7^ NJ is characterized by elevated TBIL levels in serum, which are associated with abnormalities in bilirubin intestinal and hepatic circulation. The gut microbiota plays an important role in the excretion of bilirubin.^8,9^ Research found that the serum TBIL levels in germ-free mice were higher than in non-germ-free mice.^10^ According to related research, downregulating *Bifidobacterium* species, including *B. adolescentis*, *B. bifidum*, and *B. longum*, was linked to higher serum TBIL levels. It is suggested that the gut microbiota may be involved in bilirubin metabolism.^11^ The current understanding is that the gut microbiota and hepatointestinal signal transduction directly participate in the metabolism and excretion of bilirubin. That is, unconjugated bilirubin, which is produced when red blood cells are destroyed, enters the liver first and is discharged from the liver by combining with UDP-glucuronic acid to form conjugated bilirubin (glucuronic acid ester). Most of the conjugated bilirubin excreted out of the liver enters the biliary tract, where the conjugated bilirubin is broken down by intestinal bacteria and reduced to urinary bilirubin and excreted in the feces.^12,13^

We previously conducted a metagenomic association study on neonates with breast milk jaundice and cholestatic jaundice, and found that the changes in the gut microbiota of these neonates were related to an increase in serum bilirubin. The neonates with jaundice exhibited significantly lower abundance of *Bifidobacterium* bacteria and genes related to galactose metabolism, which was negatively correlated with serum TBIL levels.^5^

It is known that *Bifidobacterium* is directly involved in the utilization of galactooligosaccharide (GOS)^14^ and converts GOS to galactose and UDP-glucose via the galactose metabolism pathway. Considering that UDP-glucose, the product of galactose metabolism, is the precursor of UDPGA, and UDPGA is directly involved in the formation of direct bilirubin, we hypothesized that the gut microbiota and metabolites may play an important role in the development of jaundice. We also studied the metabonomics of gut metabolites in neonates with jaundice and healthy controls. Based on machine learning and a causal inference approach, we found that gut metabolites can distinguish jaundice from healthy neonates, and the change in gut metabolites in neonates with jaundice showed that branched-chain amino acids were positively correlated with serum TBIL.^15^ This further confirmed that intestinal metabolites play an important role in the occurrence and development of jaundice.

An accurate disease risk prediction model is important for identifying low-risk and high-risk individuals with respect to NJ. This is due to the fact that, if neonates belong to the high-risk category, targeted screening and interventions can be provided to address their risk of disease, and if they fall into the low-risk category, unnecessary screening and intervention can be avoided. The observational relationship between suspected risk factors and results does not always indicate that the intervention of risk factors will have a causal relationship with the results (correlation is not causality).^16^ The causal inference method is used to find the potential causal relationship from the correlation results.^17^ We previously used multi-omics bioinformatics analysis of the metagenome-metabolome to capture key bacteria involved in critical glutamate metabolism in the gut microbiota of individuals with autism, and discovered the increase in the bile acid-metabolizing bacterium *Eggerthella lenta* and its interaction with glutamate metabolism.^18^ The recent development of causal science research methods has also accelerated the process of using multi-omics techniques to reveal the potential pathogenesis of complex diseases.^19,20^

In this study, we explore the bacteriome-metabolome data landscape of NJ through a “multi-omics” approach, and utilize the causal effect evaluation method of machine learning to gain a comprehensive understanding of the gut microbiota and metabolite composition that affects NJ, and to validate these findings with the expectation of discovering key bacterial and metabolite molecules for the early diagnosis of NJ.

## Methods

### Participants and sample collection

This study included 98 NJ newborns and 98 healthy control (HC) newborns. The study was divided into two stages: the discovery stage and the validation stage. In total, 68 NJ newborns and 68 HC newborns were included in the discovery stage, and 30 NJ newborns and 30 HC newborns were included in the validation stage. NJ, also known as neonatal hyperbilirubinemia, was diagnosed with reference to the American Academy of Pediatrics Intervention Guidelines for Neonatal Jaundice^21^ and the Expert Consensus on the Diagnosis and Treatment of Neonatal Hyperbilirubinemia of Neonatal Group of Neonatology of the Pediatrics Branch of the Chinese Medical Association.^22^ The inclusion and exclusion criteria are detailed in the **Supplementary Appendix**. The neonates were categorized as either NJ or HC based on their serum bilirubin level (total bilirubin, TBIL), which was measured using a Beckman Coulter automatic biochemistry analyzer during their hospitalization (Beckman Coulter, CA, USA).

The researchers wore masks and gloves, and used sterilized disposable fecal sampling tubes to collect the feces excluded from newborns after birth, so as to avoid artificial pollution. Then transported on ice overnight to our laboratory, then immediately dispensed at 3–5 g/tube and stored at ˗80°C. The hospital’s medical ethics committee approved the study methodology, which followed the Declaration of Helsinki, and the parents of each newborn provided written informed consent.

### Gut microbiome analysis

Refer to our published articles,^23–25^ for detailed methodology on 16S rRNA gene sequencing and bioinformatic analyses (detailed in the Supplementary Appendix).

### Gut metabolome analysis

Refer to previously published articles^18,26–28^ for detailed methodology on the analysis of NJ-associated gut metabolites. Fecal bile acids were detected and analyzed by targeted metabonomics and UPLC-QQ-MS/MS (detailed in the Supplementary Appendix).

### Gut microbiome-metabolome association

#### Network module analysis

First, the corAndPvalue function in the R package WGNCA (version 1.72–1) was used to calculate the correlation coefficient of species. Then, the R-package multitest (version 2.54.0) was used to correct the statistically significant *p* value derived from the Benjamini – Hochberg procedure, and the corrected *p* value was less than 0.001 and the absolute value of the correlation coefficient was greater than 0.8, which is defined as a significant co-occurrence network. Then, the R package igraph (version 1.5.1) was used to visualize the network structure. Finally, the linear correlation analysis of species abundance with clinical variables and metabolites of the top four network modules was completed by the geom_smooth and stat_cor functions in ggplot2 (version 3.4.4) of the R package.

#### Mantel test

First, based on all or well-grouped species variables and environmental variables matrices, the Mantel_test function in R package linkET (version 0.0.7.4) was used to perform a mantel test to determine the correlation between the two matrices. Then, the correlation function in R package linkET was used to determine the correlation coefficient matrix between environmental variables. Finally, qcorrplot in the R package linkET was used to visually show the correlation between the two matrices.

#### Procrustes analysis

Procrustes analysis is a method of comparing the consistency of two sets of data by analyzing the shape distribution. The principle is least-squares orthogonal mapping, which means finding the canonical shape by constant iteration, and using the least-squares method to determine the affine variation in each object shape compared with the standard shape. Procrustes analysis was completed using the R package vegan, with reference to a previous publication.^29^ First, principal component analysis (PCA) dimensionality reduction was performed through the rda function on the two datasets separately and the coordinates of the feature axes (which represent linear combinations of the sets of variables) were extracted for comparison. Then, through the procrustes function, and using the parameters: permutations = how, nperm = 999, we obtained the deviation sum of squares M2 statistic and the *p* value after a 999 permutations test. Finally, images were displayed through the R package ggplot2.

#### Survival analysis

A Kaplan – Meier survival curve was constructed using the KaplanMeierFitter function of the Lifelines package (version 0.26.4) of Python software (version Python 3.7.6). When conducting univariate survival analysis, we first set up the time variable and the event variable, then compared the differences. Statistical significance was achieved by the logrank_test function of the statistics module of the lifelines package, and a *p* value of less than 0.05 was regarded as significant. Multivariate survival analysis was conducted through the lifelines package CoxPHFittert function.

### Causal inference analysis

#### Causal mediation analysis

The principle of causal mediation analysis was to identify and explain the causal link between the independent variable (X) and the dependent variable (Y) by introducing a mediating variable (M). We carried out causal mediation analysis as described in the Supplementary Appendix, in accordance with our earlier research.^28^

#### Structural equation model (SEM)

First, based on either gut microbiota genus level data (profile) or gut bile acid data (profile), the vegdist and pcoa functions in the R package Vegan (version 2.6–4) were used to count the Bray – Curtis distances and perform principal co-ordinates analysis (PCoA), respectively, and the resulting first axis (PC1) was used to represent the microbial community or bile acid beta-diversity. Second, for the data of PC1 and the variables to be studied, the scale in the R package base (version 4.2.3) was used to standardize the data, and a set of structural equations of “Y~X” was constructed. The sem function in the R package lavaan (0.6–16) was used to fit the model. The parameter estimates function in the R package lavaan was used to obtain the model’s partial regression coefficients (estimate), the execution intervals, and the *p* value. The fitMeasures function in the R package lavaan was used to assess the reliability of the model parameters, with *p* > 0.05 implying the reliability of the model, i.e., the model predictions were not significantly different from the actual observations. Finally, the semPaths function in the R package semPlot (version 1.1.6) provided an alternate visual presentation of the structural equation model.

#### Machine learning-causal inference

As per our prior research studies^16,30^ and as explained in the Supplementary Appendix, causal inference based on machine learning was completed through the Microsoft DoWhy library (https://github.com/microsoft/dowhy.) and the EconML library (https://github.com/econml/).

#### Clinical predictive modeling evaluation

A clinical prediction model for NJ was constructed through accuracy assessment, clinical effect assessment, and risk prediction (as detailed in the Supplementary Appendix).

#### Machine learning models

Lasso machine learning, the lasso+xgboost model, and the random forest model were adopted. For details, refer to previously published articles^27,31^ and the Supplementary Appendix.

### Other analysis

To evaluate the correlation between the clinical manifestations and gut microbiota composition/metabolites with significant differences between groups, the lm function in the R software was used to construct the logistic regression model, and the *p* value and coefficient of determination (R-squared) of the logistic regression model were obtained through the summary function. The R package beeswarm was used to draw boxplots and scatterplots, and *p* values with significant differences were obtained by referring to the wilcox.test function. A ridgeline plot was completed using the R package ggplot2 and a personalized script. Species diversity analysis (Shannon index) and radar diagrams were constructed using personalized scripts. The correlation heatmap was completed by the labeledHeatmap function in the R package WGCNA, and significance was set to an absolute value of Spearman’s correlation coefficient greater than 0.3 and a *p* value less than 0.05.

## Results

### Participant information and composition

**Figure 1.**
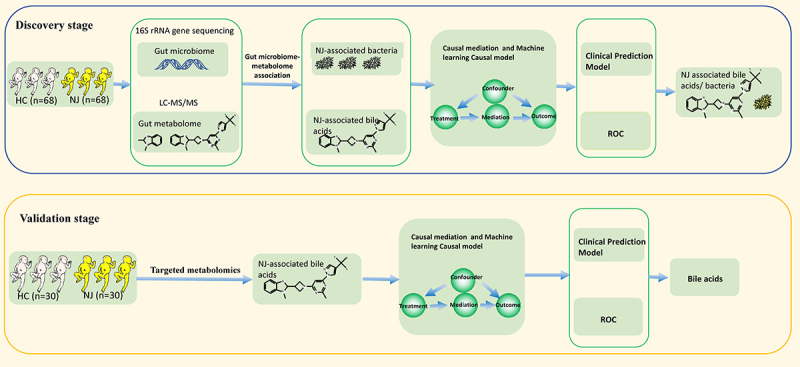
Flow chart of the study design. A total of 98 neonates with neonatal jaundice (NJ) and 98 healthy control (HC) neonates were included in this study, which was divided into two stages: the discovery stage and the validation stage. We collected initial feces samples from 68 neonates with NJ and 68 HC neonates in the discovery stage, and 30 neonates with NJ and 30 HC newborns in the validation stage. In the discovery stage, 16S rRNA gene sequencing technology was used to obtain the gut microbiota composition of each sample, and with the same batch of samples, liquid chromatography with tandem mass spectrometry (LC-MS/MS) was used to obtain the metabolome composition of each sample. Then, gut microbiota association analysis was used to obtain the nj-associated gut microbiota composition, while metabolome association analysis was used to obtain the nj-associated metabolite composition. Gut microbiome-metabolome association analysis was employed to discover NJ/clinical indices-associated gut bacteria and bile acids. To further understand the association of gut bacteria/bile acids with NJ, we assessed the key bacteria and bile acids with causal effects on NJ/clinical indices based on causal mediation analysis. Then, we constructed a causal model based on a machine learning-causal inference method. Finally, clinical prediction models based on gut bacteria and bile acids were constructed and used for clinical effect assessment and risk prediction, while random forest machine learning methods were used to assess the clinical diagnostic potential of gut bacteria and bile acids. In the validation stage, we used targeted metabonomics detection to determine the composition of intestinal metabolites. The machine learning method was used to evaluate the importance of metabolites in the classification of NJ, and a clinical prediction model was constructed for NJ based on intestinal metabolites.

**Figure 2.**
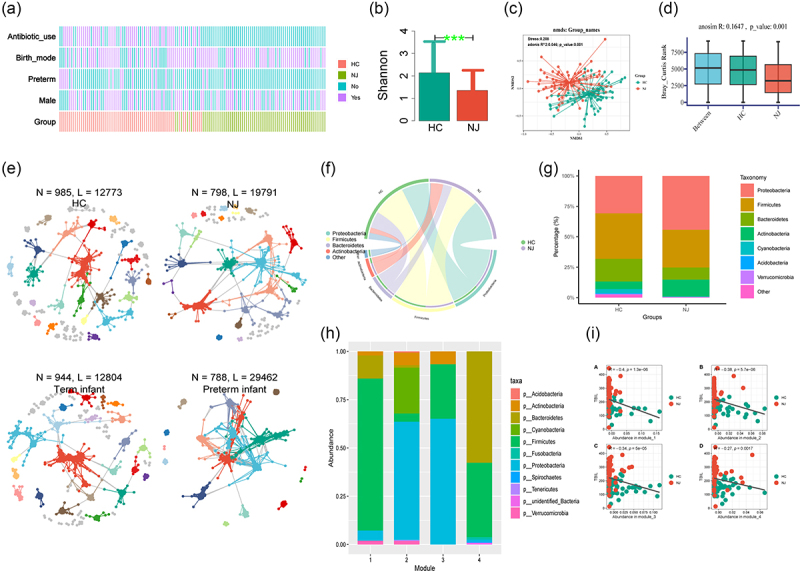
Nj-associated gut bacteria network module. (a) Comparison of clinical phenotypes between the NJ and HC groups; (b) diversity of fecal microorganisms in both groups. The vertical coordinate represents the Shannon index, with larger values indicating a higher response biodiversity (i.e., more species indicates that the samples were more evenly distributed). The gut microbiota diversity was significantly lower in neonates with jaundice than in the controls, ***p value < 0.001; (c) NMDS analyses between the NJ and HC groups; (d) anosim analysis showed differences between the two subgroups (*R* = 0.1647, *p* = 0.001); (e) visualization of molecular ecological networks (MENs) constructed based on the structure of the gut microbiota. Large modules with ≥5 nodes are shown in different colors, and smaller modules are shown in gray; (f) the gut microbiota composition of the NJ and HC groups as shown in chord diagrams; (g) relative abundance of the gut microbiota in the NJ and HC groups at the phylum level; (h) module diagram based on the construction of the gut microbiota; (i) comparison of the relationship between modules with different gut microbiota abundances and TBIL levels.

This study included 98 NJ and 98 HC neonates. Of these, 68 NJ and 68 HC neonates were included in the discovery stage, and 30 NJ and 30 HC neonates were included in the validation stage. The overall study design is shown in Figure 1. Comparison of the characteristics between the NJ and HC groups in the discovery stage, revealed 73 males (38 in the NJ group and 35 in the HCs), 20 preterm newborns (4 in the NJ group and 16 in the HCs), and 99 vaginal deliveries (55 in the NJ group and 44 in the HCs), as shown in Figure 2(a). The statistical analyses was shown in Supplementary Appendix.

### NJ-associated gut bacteria network module

Comparing the gut microbial diversities between the groups revealed significantly lower Shannon diversity indices in the NJ group than in HCs (Figure 2(b)). Non-metric multidimensional scaling (NMDS) analysis showed significant clustering between the intestinal flora of the NJ and HC groups (Figure 2(c)). Anosim analysis confirmed differences between the two subgroups (Figure 2(d)). Then, we used 16S rRNA sequencing-based molecular ecological networks (MENs) and visualization tools to reveal the interrelationships among gut microbes between groups. The results revealed a gut microbial interaction network of 798 nodes (operational taxonomic units, OTUs) and 19,791 links (interactions) for the NJ group, compared with 788 nodes and 29,462 links (interactions) for the preterm infant group. While more nodes but fewer links were observed in the networks constructed by the HC and term infant groups (Figure 2(e)). Based on the differences in sex and the use of antibiotics, we also used MENs to reveal the relationship between the two groups of intestinal microorganisms. The results showed that in the antibiotic use group, fewer nodes but more links were observed. By contrast, more nodes but fewer links were observed for male newborns than female newborns (Figure S1A). We used a chord diagram to visually analyze the gut microbial composition between the NJ and HC groups, and both groups were found to be dominated by the phyla Firmicutes and Proteobacteria (Figure 2(f)). Then, we further analyzed the gut microbiota composition between the NJ and HC groups at the phylum level, and a higher percentage of Proteobacteria was detected in the NJ group than the HC group, and a lower ratio of Firmicutes was detected in the NJ group than the HC group (Figure 2(g)). The different gut microbiotas were then categorized into four modules based on their abundance (Figure 2(h)), with the highest abundance of Firmicutes in Module 1, the highest abundance of Proteobacteria in Modules 2 and 3, and the highest abundance of Bacteroidetes and Firmicutes in Module 4. Then, we compared the relationship between modules with different gut microbiota abundance and TBIL, and a significant negative correlation was detected between all of the modules and TBIL (Figure 2(i)).

### Gut microbiome-metabolome association analysis

In the discovery stage, considering the same batch of samples, we determined the composition of the gut microbiota by 16S rRNA gene sequencing and determined the composition of metabolites by metabolomics detection by liquid chromatography and tandem mass spectrometry (LC-MS/MS). We then performed gut microbiome-metabolome association analysis.

Based on the microbiome association analysis, we identified 68 significantly different members of the gut microbiota based on composition, the vast majority of which (59/68) were enriched in the HC group. Only nine bacteria showed significant enrichment in the NJ group, namely *Erysipelotrichaceae*, *Erysipelotrichales*, *Erysipelotrichia*, *Escherichia coli*, *Gammaproteobacteria*, *Staphylococcaceae*, *Staphylococcus*, *Staphylococcus warneri*, and unidentified *Enterobacteriaceae* (Table S1). Association analysis based on metabonomics revealed 48 different compositions of gut metabolites, most of which (28/48) were enriched in the HC group, with 20 metabolites significantly enriched in the NJ group (Table S2).

**Figure 3.**
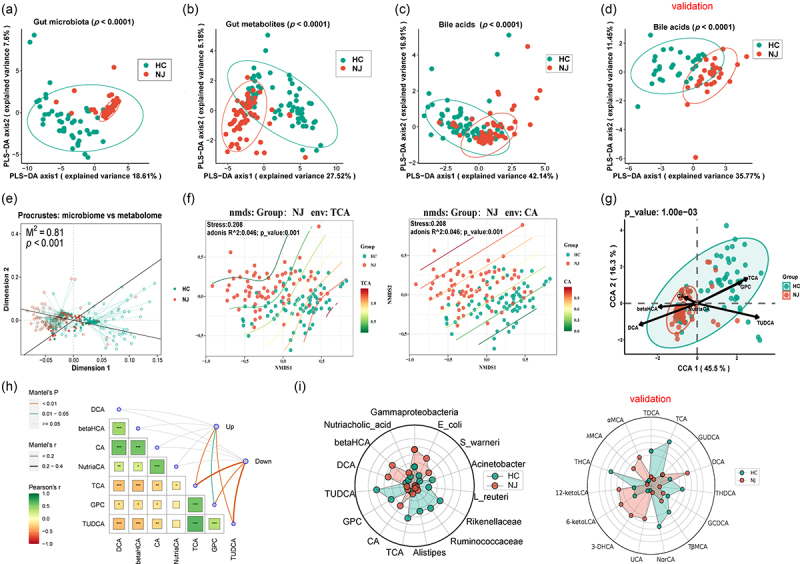
Analysis of the nj-associated gut microbiota and metabolites. (a) Results of PLSDA analysis indicate that the nj-associated gut microbiota can distinguish between the NJ and HC groups to some extent; (b) results of PLSDA analysis indicate that nj-associated gut metabolites can distinguish between the NJ and HC groups; (c,d) results of PLSDA analysis indicate that nj-associated gut bile acids can distinguish between the NJ and HC groups in the discovery and validation stages; (e) a strong correlation between nj-associated gut microbiota composition and metabolite composition was found by procrustes analysis; (f) NMDS analysis shows significant clustering of intestinal bile acids TCA and CA between the NJ and HC groups; (g) correlations between bile acids and genus-level gut microbiota, as determined by CCA analysis; (H) mantel analysis shows a strong correlation between intestinal bacteria and bile acids; (I) a radar chart reveals differences in the contents of intestinal bacteria and bile acids between the two groups in the discovery and validation stages.

We assessed whether the NJ-associated gut microbiota could distinguish between the NJ and HC groups by PLSDA analysis, and the results showed that it was able to distinguish between the groups to some extent. Plsda1 could explain 18.61% of the compositional variance of the gut microbiota and the samples were well separated along the plsda1 axis, whereas plsda2 could explain 7.6% of the gut microbiota compositional variation (Figure 3(a)). Additionally, we evaluated whether NJ-associated gut metabolites could distinguish between the NJ and HC groups by PLSDA analysis, and the results demonstrated that they were able to distinguish between the groups. Plsda1 and plsda2 explained 27.52% and 5.18% of the variation in the gut microbiota composition, respectively, and the samples were well separated along the plsda1 axis (Figure 3(b)). Then, we evaluated whether NJ-associated gut bile acids could distinguish between the NJ and HC groups by PLSDA analysis, and the results demonstrated that they could distinguish between the groups. Plsda1 and plsda2 explained 42.14% and 16.91% of the variation in the gut microbiota composition in the discovery stage, respectively (Figure 3(c)), and 35.77% and 11.45% of the variation in the gut microbiota composition in the validation stage, respectively (Figure 3(d)). Furthermore, we used Procrustes analysis, a method of assessing the consistency of two sets of data via analysis of the shape distribution, to further understand the association between NJ-associated changes in gut metabolites and the NJ-associated gut microbiota. We found that NJ-associated gut microbiota composition and metabolite composition were strongly correlated (Figure 3(e)).

To further asses the differences in the gut bile acids between the two groups, we used NMDS analysis of the Bray – Curtis distance matrix generated from genus-level abundance. This revealed a significant difference in the intestinal bile acids TCA and CA between the NJ and HC groups at the genus level (*p* < 0.0001) (Figure 3(f)), as well as significant differences in the bile acids TUDCA, DCA, NutriaCA, and βHCA between the two groups (Figure S1B).

Gut microbiota may be more relevant to bile acid metabolites, so bile acid metabolites were used as environmental variables and correlations between bile acids and the gut microbiota were found by canonical correspondence analysis (CCA), as shown in Figure 3(g). Redundancy analysis (RDA) revealed a correlation between the gut microbiota and bile acid markers (Figure S1C). Mantel analysis of all gut bacteria with bile acid markers confirmed a significant correlation (Figure S1D), and furthermore revealed a strong correlation between intestinal bacteria markers and bile acids (Figure 3(h)). We further compared each bile acid level between the two groups and found that there was a significant difference in bile acids between the two groups, as shown in Figure S2. The radar chart revealed that there were differences in the contents of intestinal bacteria and bile acids between the two groups in the discovery and validation stages (Figure 3(i)).

The length of an environmental parameter arrow indicates the strength of the environmental parameter with regard to the overall gut microbiota. The results showed that there was a significant correlation between these seven bile acids and the gut microbiota. We performed a biological pathway enrichment study on NJ-associated metabolites to gain a deeper understanding of their biological significance. The results showed enrichment of the pathway linked to bile acids (Figure S3A). We also carried out enrichment analysis according to the type of disease, and found that enrichment was linked to recurrent *Clostridium difficile* infection, with the metabolites involved being the metabolites of bile acids (Figure S3B).

To further understand the correlation between NJ-associated gut metabolites and bile acids, we performed Procrustes analysis and found a strong association between NJ-associated gut metabolite composition and bile acids (Figure S4A). We further analyzed the correlation between NJ-associated gut bacteria and bile acids, and the results confirmed this correlation (Figure S4B).

### Machine learning approach discovers bacteria associated with bile acids

Considering that NJ-related metabolites are enriched to bile acid-related pathways, we next focused on bile acid metabolites and investigated bacteria associated with bile acid metabolites by the Lasso machine learning method. Among the enterobacteria, *Lactobacillus reuteri* had a positive effect on gut taurodeoxycholic acid (Figure S4C), *Rikenellaceae* and *Ruminococcaceae* had a negative effect on gut 1beta hydroxycholic acid, and *Staphylococcus* had a positive effect on 1beta hydroxycholic acid (Figure S4D). The enterobacteria positively affecting gut butylcholic acid were *Gammaproteobacteria* and *Erysipelotrichales*, whereas *Ruminococcaceae* had a negative effect on cholic acid (Figure S4E). The gut bacterium positively affecting deoxycholic acid was *Gammaproteobacteria*, whereas *Rikenellaceae* and *Ruminococcaceae* had a negative effect on deoxycholic acid (Figure S4F). Two other bile-related acid derivatives, glycerophosphocholine and nutriacholic acid, were also affected by intestinal bacteria, with *Alistipes* and *Acidobacteria* positively affecting intestinal glycerophosphocholine (Figure S4G) and *E. coli* positively affecting nutriacholic acid (Figure S4H).

Further, we evaluated the potential of Lasso machine learning to assess the gut microbiota abundance for predicting the content of gut bile acids, and found that in addition to the content of gut bile acids 1beta hydroxycholic acid and nutriacholic acid being predicted based on the gut bacteria (Figure S4I, J), NJ can also be predicted based on the gut bacteria (considering NJ to be 1 and HC to be 0) (Figure S4K). We focused on five bacteria (*Acinetobacter*, *Alistipes*, *L. reuteri*, *Rikenellaceae*, *Ruminococcaceae*) (Figure S5A – E) with significant decreases in abundance in the NJ group and one bacterium that significantly increased in abundance in the NJ group (*Gammaproteobacteria*) (Figure S5F), to learn more about the connection between bile acids and the intestinal bacteria linked with NJ.

Using the networkx software for network relationship analysis, it was possible to observe the complicated network link between the bacteria in the gut and bile acids associated with NJ in the discovery and validation stages (Figure S6A, B).

We also assessed the linear relationship between NJ-associated gut microbiota and bile acids. It was discovered that the quantity of intestinal bacterial *Rikenellaceae* and the content of intestinal bile acid (taurocholic acid) and its product, glycerophosphocholine, were positively correlated. The abundance of enteric bacteria *Alistipes* was positively correlated with gut bile acid derivative glycerophosphocholine, and the gut bile acid taurodeoxycholic acid showed a positive relationship with the abundance of gut bacterium *L. reuteri* (Figure S6C – F).

### NJ-associated bacterial/bile acid metabolites correlate with serum TBIL levels

**Figure 4.**
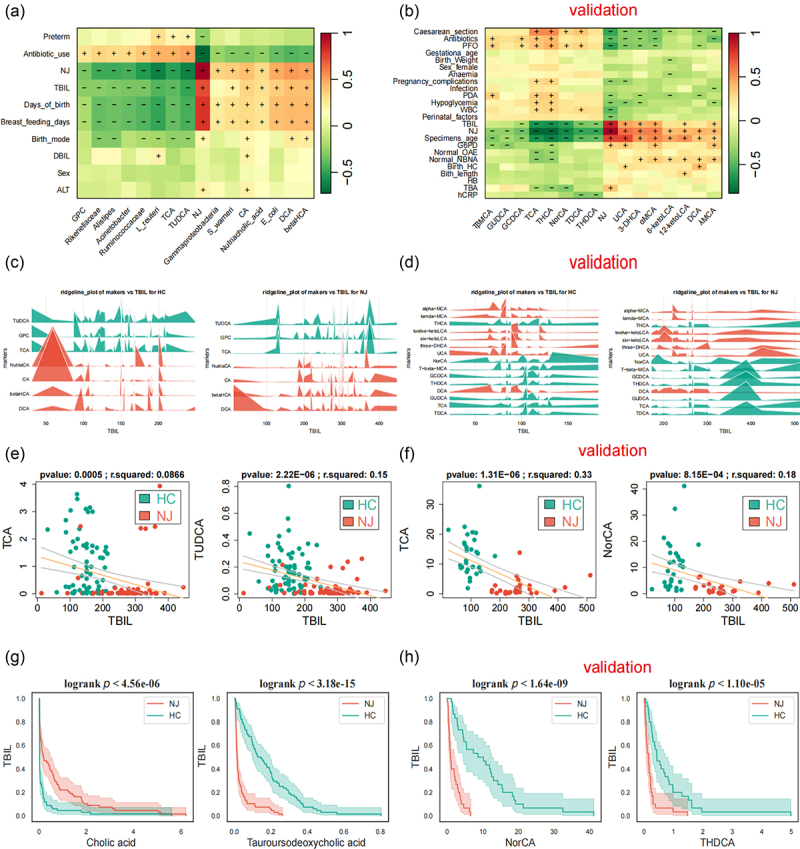
Correlation between nj-related intestinal bacteria and bile acids and clinical phenotype. (a,b) correlation heatmap between nj-related intestinal bacteria and bile acids and clinical phenotype in the discovery (a) and validation (b) phases; (c,d) nj-related bile acids are correlated with TBIL in the discovery (c) and validation (d) phases; (e) nj-related bile acid TCA and bile acid derivative glycerophosphocholine were negatively correlated with TBIL in the discovery stage; (f) nj-related bile acid TCA was negatively correlated with TBIL in the validation stage; (g,h) survival analysis to assess the relationship between nj-associated gut bacteria and bile acid metabolites and TBIL in the discovery (g) and validation (H) stages.

To understand the clinical significance of NJ-associated bacterial and bile acid metabolites, we performed a heatmap correlation analysis of NJ-associated gut bacterial/bile acid metabolites with clinical indicators in the discovery and validation stages (Figure 4(a,b)). The results showed that NJ-associated gut bacteria/bile acids were positively/negatively correlated with TBIL to varying degrees. At the same time, we analyzed the content of bile acids at different TBIL levels using a ridgeline plot (Figure 4(c,d)). We also analyzed the distribution of bile acids in the NJ and HC groups at different breastfeeding durations and at different white blood cell levels using a ridgeline plot (Figure S7A, B).

Then, we correlated NJ-associated bile acid metabolites with clinical indicators and the results showed that the NJ-associated intestinal primary bile acid taurocholic acid and the bile acid derivative glycerophosphocholine were significantly negatively correlated with serum TBIL levels (Figure 4(e)). Moreover, there was a negative relationship between serum TBIL levels and the secondary bile acid tauroursodeoxycholic acid. Meanwhile, we correlated the abundance of gut bacteria with clinical indicators and showed that the abundance of gut bacteria *Acinetobacter*, *Alistipes*, *Rikenellaceae*, and *Ruminococcaceae*, which affect bile acid levels, was negatively correlated with serum TBIL levels (Figure S7C). We also analyzed other clinical indicators, which showed that gut bacteria and bile acid levels were less affected by mode of delivery between groups with different modes of delivery in the discovery and validation stage (Figure S8).

We assessed the relationship between TBIL and NJ-associated intestinal bacteria and metabolites using survival analysis, and discovered that there was a significant difference in the increase in TBIL between the NJ and HC groups, with the NJ group experiencing a greater increase as the abundance of the intestinal bacteria *Acinetobacter*, *L. reuteri*, and *Rikenellaceae* decreased. With the rise in the gut bile acid derivative nutriacholic acid, the increase in TBIL was significantly different between the NJ and HC groups, with a greater increase in the NJ group (Figure S9A). As the intestinal primary bile acid cholic acid increased and the secondary bile acid tauroursodeoxycholic acid decreased, there was a significant difference in the magnitude of the rise in TBIL between the NJ and HC groups, with a greater rise in the NJ group (Figure 4G). In the validation stage, as the gut bile acids NorCA and THDCA decreased, there was a significant difference in the magnitude of the rise in TBIL between the NJ and HC groups, with a greater rise in the NJ group (Figure 4(h)). As the intestinal bile acids THDCA, THCA, TDCH, and TβMCA declined, the difference in the magnitude of TBIL increase between the NJ and HC groups was significant, with a greater increase in the NJ group (Figure S9B).

### Potential causal effects of NJ-associated bacterial/bile acid metabolites with NJ

Considering the correlation between NJ-associated bacteria/bile acid metabolites and serum TBIL levels of NJ and clinical indicators, we used causal mediator analysis to assess the causal relationships between bile acid-associated bacteria, bile acids, and core clinical indicators to better understand whether NJ-associated bacteria impact on bile acid metabolism, serum TBIL levels, and clinical indicators.

**Figure 5.**
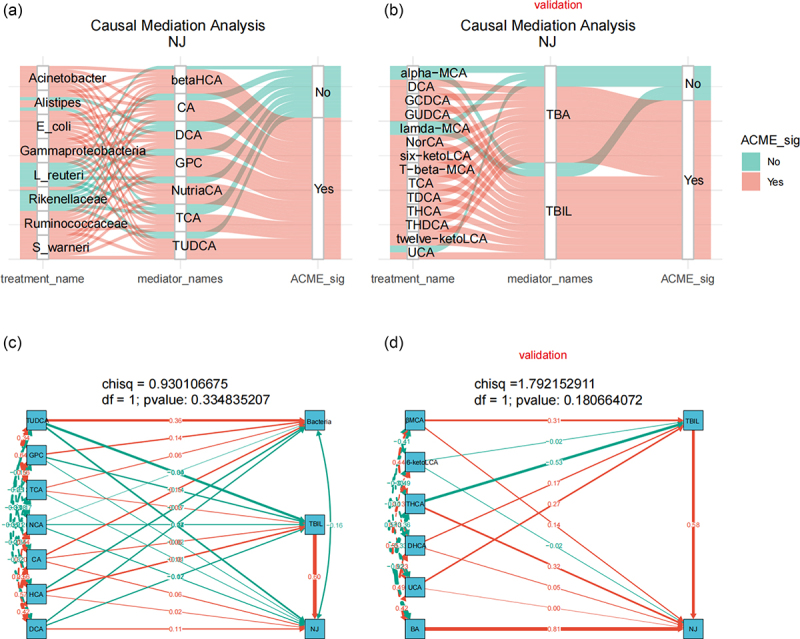
Assessment of the potential causal effects of bile acid-related bacteria and bile acids with NJ and clinical indicators. (a,b) causal inference analysis indicates that gut bacteria affect NJ by influencing bile acid levels in the discovery stage (a), and bile acids affect NJ by influencing TBIL levels in the validation stage (b); (c,d) the causal inference model was tested by SEM and found to be reliable (*p* > 0.05 indicates that the model is reliable).

The results of the causal mediator analysis showed that intestinal bacteria not only affect the concentrations of TBIL, serum direct bilirubin (DBIL), and alanine aminotransferase (ALT) (Figure S10), which are the clinical markers of NJ, but also affect NJ itself by affecting the levels of bile acids (Figure 5(a)). Furthermore, intestinal bile acid metabolites affect NJ by influencing TBIL levels in the validation stage (Figure 5(b)). We further tested the causal model using structural equation modeling (SEM), and a *p* value > 0.05 suggested that the model was reliable, as shown in Figure 5(c,d).

### Construction of a clinical prediction model based on NJ-related bacterial/bile acid metabolites

**Figure 6.**
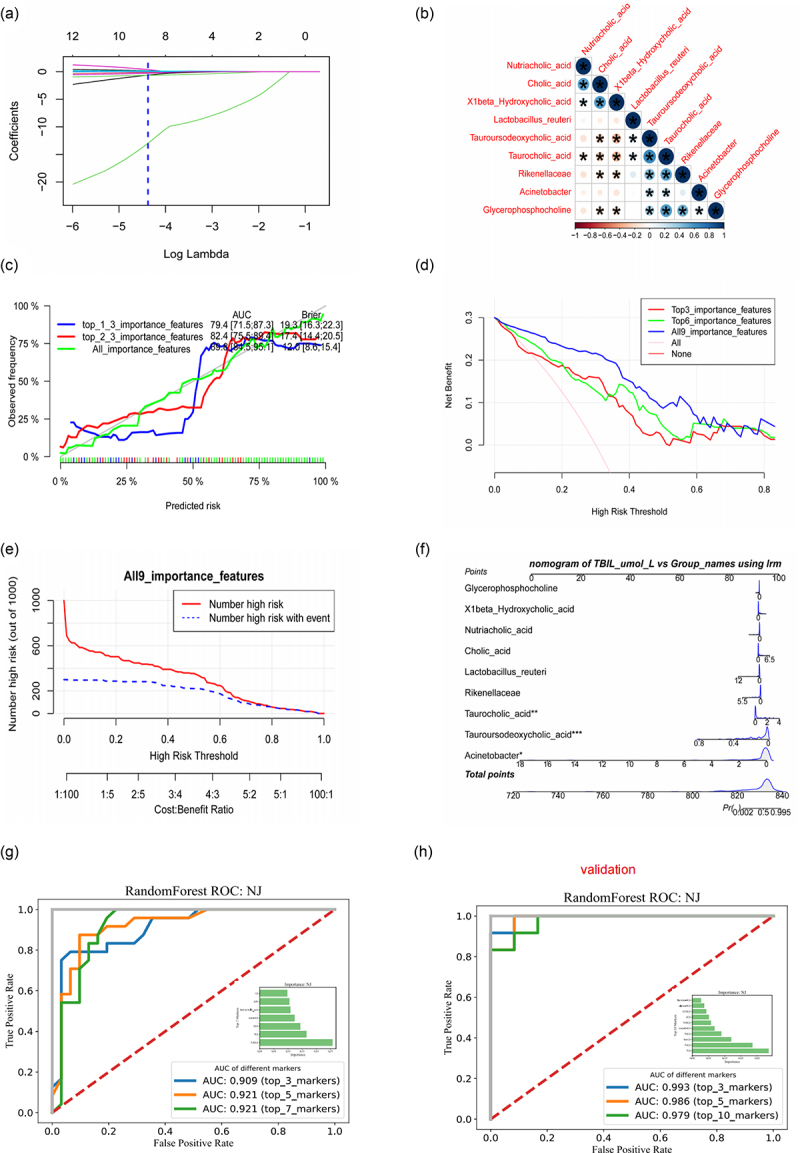
Construction of a clinical prediction model for jaundice. (a) LASSO machine learning method to filter the optimal variables for NJ classification; (b) heatmap of the correlation of important variables, the asterisk represents a significant correlation; (c) calibration curve, the calibration of the clinical prediction model, is an important indicator to evaluate the accuracy of a disease risk model to predict the probability of an outcome event in an individual in the future; (d) the horizontal coordinate is the threshold probability, when various evaluation methods reach a certain value, the probability of NJ in patient i is recorded as pi, and when pi reaches a certain threshold (recorded as Pt), it is defined as positive and some intervention is taken (such as changing the blue light treatment plan). Changing the treatment regimen naturally changes the balance between positive and negative clinical indicators and severe jaundice, and the vertical coordinate is the net benefit (NB) after subtracting the positives from the negatives. It can be seen that the clinical decision model consisting of important variables has some clinical effect; (e) clinical impact curve, using a model consisting of all variables to predict the risk stratification of 1,000 individuals, showing the “loss:benefit” axis, assigned to eight scales, the red curve (number of high risk) indicates the risk stratification at each blue curve (number of high risk with outcome) is the number of true positives at each threshold probability. From the graph it can be seen that intervention at a threshold of ≤ 0.6 can reduce injury and increase benefit. The number of individuals classified as positive (high risk) by the model under the rate; (f) the risk of NJ occurrence can be predicted based on the clinical prediction model; (g,h) nj-associated bile acids are potential biomarkers for NJ in the discovery and validation stages.

To understand the potential clinical applications of NJ-related bacterial/bile acid metabolites, we further screened gut bacterial and bile acid variables affecting NJ using Lasso machine learning. We found nine significant variables (Figure 6(a)), including three bacteria and six bile acids (Figure 6(b)). The optimal model formulas and evaluations are shown in Figure S11A. Taurocholic acid, *Acinetobacter*, and tauroursodeoxycholic acid had the most significant impact on the model.

Based on the nine important variables, we constructed clinical prediction models for the top three important variables (top3), the top six important variables (top6), and all important variables (all), considering the ranking of important variables obtained based on Lasso analysis. To assess whether the model-predicted risks were in good agreement with the actual occurrence risks, we performed calibration of the clinical prediction models and found that all models predicted risks in good agreement with the actual occurrence risks. The clinical prediction models based on all important variables (all) had the highest Brier scores and AUCs (Figure 6(c)). Given that determining whether a patient has a particular disease using a particular biomarker will inevitably result in false positives and false negatives, depending on the situation, it is sometimes preferable to avoid false positives and sometimes preferable to avoid false negatives.

Since neither situation can be avoided, we tried to find a model with the largest net benefit by decision curve analysis (DCA). As shown in Figure 6(d), clinical decision model based on the composition of all important clinical variables has a certain clinical effect (net benefit). By using the clinical impact curve to assess the clinical effect of the model with all variables included, we were able to determine that intervening at a threshold of ≤ 0.6 can decrease damage and improve the benefit (Figure 6(e)). After building a logistic regression model for NJ prediction of risk by considering all variables, we were able to display the model using a column-line diagram (Figure 6(f)). This demonstrated that the model was more accurate in predicting the risk of severe NJ (SFigure S11B, SC).

To evaluate the potential of gut bile acids for the early diagnosis of NJ, we constructed classifiers based on the random forest model. The first three intestinal bile acids (i.e., TUDCA, TCA, DCA) had an AUC value of 0.909 for NJ classification and the AUC value for the first five intestinal bile acids used for NJ classification was 0.921, suggesting that gut bile acids are potential biomarkers for NJ (Figure 6(g)). To evaluate the potential of intestinal bile acids for the early diagnosis of NJ, a classifier was constructed based on the random forest model. The AUC value of the first three bile acids (i.e., TCA, THCA, NorCA) for NJ classification was 0.993 (Figure 6(h)), suggesting that intestinal bile acids are potential biomarkers of NJ.

## Discussion

It is uncertain how early-life interactions between the human gut microbiota and the metabolome contribute to human disease. It is essential to gain a comprehensive understanding of the bacteria engaged in bile acid metabolism in the gut since the gut microbiota is both impacted by and implicated in bile acid metabolism. Bile acids are steroid molecules derived from cholesterol that play an important role in energy balance, host metabolism, and maintenance of innate immunity through G protein-coupled receptors and/or nuclear receptors.^32^ There are complex interactions between the gut microbiota and bile acids. Bile acids promote the growth of bile acid-metabolizing bacteria and inhibit the growth of other bile-sensitive bacteria to reshape the gut microbiota. Additionally, the gut microbiota can modify primary bile acids into secondary bile acids by producing a variety of enzymes, such as bile salt hydrolase and hydroxysteroid dehydrogenase, to influence the metabolism of bile acids and the composition of the bile acid pool, further degrading the bile acids by other enzymatic mechanisms, thus helping to maintain cholesterol homeostasis.^33^

In this study, we investigated the composition of the gut microbiota during NJ by means of a network visualization and analysis method. We discovered that the composition of the intestinal flora of jaundiced newborns was significantly less diverse than that of HC neonates.

It is well-established that the gut microbiota plays an important role in human health by participating in bile acid metabolism. We identified the NJ-associated gut microbiota and metabolite composition by gut microbiome-metabolome association analysis, and found that the NJ-associated gut microbial composition is closely related to metabolite composition.

It is a challenging task to identify the important gut microbes, metabolites, and phenotypic characteristics from high-throughput multi-omics data, such as microbiome and metabolome data, and host phenotypic characteristics. In this study, we identified NJ-associated gut microbiota and metabolites by gut microbiome-metabolome association analysis. The gut microbiota is directly involved in the process of bile acid metabolism. It is known that cholesterol is catalyzed by hydroxylase in the liver to produce primary bile acids, which are transformed into secondary bile acids under the action of intestinal bacteria after entering the intestine. The majority (>95%) of bile acids in the intestine can be reabsorbed back into the liver through the enterohepatic circulation, and only a small proportion (5%) are excreted through feces.^23^ Alterations in the structure and function of the intestinal flora may directly affect the enterohepatic circulation of bile acids. We found that the decreased abundance of bacteria such as *Acinetobacter*, *L. reuteri*, and *Rikenellaceae* in the intestinal tract of newborns with NJ was closely associated with abnormalities in bile acid metabolism. Related studies have found that disturbances in bile acid metabolism mediated by the gut microbiota play an important role in human liver disease. An altered gut microbiota and bile acid composition in patients with primary sclerosing cholangitis, and loss of negative feedback control of bile acid synthesis mediated by intestinal flora leads to increased hepatic bile acid concentrations and disruption of bile duct barrier function, which lead to fatal liver damage.^34^ Studies have demonstrated functional interactions between bile acid composition, gut microbiota, and metabolic phenotypes.

We constructed a clinical prediction model for NJ based on machine learning, which enabled the accurate prediction of high-risk individuals. Gut microbiome-metabolome association analysis identifies many gut microbiota and metabolites related to NJ; however, the challenge is filtering out the important variables. Lasso offers advantages in the screening of important clinical variables related to disease. Our previous study was based on the LASSO method combined with metabonomic analysis of serum and cerebrospinal fluid and we identified metabolic markers associated with neonatal sepsis in meningoencephalitis.^27^ In the current study, we employed the LASSO machine learning approach and identified nine variables that contribute significantly to NJ. Notably, five of these variables correlate closely with bile acid metabolism. Furthermore, a clinical prediction model utilizing these nine clinical variables was developed and showed promising clinical effects in accurately predicting high-risk individuals for NJ.

Through a machine learning-causal inference approach, we found that gut bacteria affected TBIL levels and NJ by affecting bile acid metabolism. Human intestinal microorganisms may encode enzymes involved in bilirubin metabolism,^35^ thereby reducing bilirubin to urobilinogen and promoting its excretion. In addition, gut microbiota can also produce β-glucuronidase, which converts conjugated bilirubin into free bilirubin, leading to an increase in free bilirubin levels and thus affecting the occurrence of neonatal hyperbilirubinemia.^36,37^ In addition, intestinal microorganisms can also produce hydrolytic enzymes that act on bile acid metabolism. It can be seen that gut microbiota may affect bile acid metabolism and bilirubin metabolism by affecting the activity of microbial enzymes, and there is a certain correlation between them. However, a correlation does not necessarily imply causation, and evidence of causality usually requires a combination of animal models or clinical randomized controlled trials, which are time-consuming and laborious. However, newly developed machine learning methods can be used to identify potential causal relationships from correlation results. We have previously used the causal inference methods of machine learning to identify oral bacteria with a potential causal connection to autism from oral microbiome data on autism.^30^

The innovation in our study is that, first, we utilized a microbiome-metabolome multi-omics approach as opposed to a single-omics approach, as well as causal mediation analysis and a causal inference method of machine learning to target the composition of the gut microbiota that influences the metabolism of bile acids. However, our study had some limitations. Considering the limited funds available, we used 16S rRNA gene sequencing but were unable to obtain the functional composition of the gut microbiota, especially the bile acid metabolizing enzyme gene composition. Furthermore, validation of the gut microbiota was lacking, and the causal model requires further validation and functional research, which will be addressed in future studies.

## Conclusion

Gut microbiome-metabolome association analysis revealed the gut microbiota and metabolite compositions associated with NJ. NJ is characterized by abnormal bile acid metabolism and is affected by the decreased abundance of gut bacteria such as *Acinetobacter*, *L. reuteri*, and *Rikenellaceae*. NJ-associated gut bacteria and bile acids are potential biomarkers of NJ, and the clinical prediction model developed in this study has certain clinical effects and can be used to predict disease risk.

## Supplementary Material

Supplemental Material

## Data Availability

The original data of the manuscript and the scripts used for data analysis will be made public after the manuscript is accepted.
