# Supplementary material for: Machine learning-causal inference based on multi-omics data reveals the association of altered gut bacteria and bile acid metabolism with neonatal jaundice
Source: Gut Microbes. 2024 Aug 21;16(1):2388805. doi: 10.1080/19490976.2024.2388805 (PMC11340767; doi:10.1080/19490976.2024.2388805)
Supplement: Supplemental Material [file KGMI_A_2388805_SM7989.zip › Supplementary_information_new clean.docx]

# Supplementary Appendix

**1 Participants and sample collection**

Inclusion criteria included that the mothers had no high-risk factors before birth, the mother did not use antibiotics, and no fetal defecation after birth before enrollment. The exclusion criteria included congenital abnormalities confirmed to exist after admission, moms with high-risk characteristics, recent antibiotic usage, neonates born before gestational age, and newborns with severe illnesses.

**2 Gut microbiome analysis**

**The 16S rRNA gene sequencing.** Initially, the extracted fecal DNA was diluted to 1 ng/μl as template DNA and polymerase chain reaction (PCR) was performed using universal primers 515F (5'-GTG CCA GCM GCC GCG GTA A-3') and 806R (5'-GGA CTA CNN GGG TAT CTA AT-3') in the V4 region of the bacterial 16S rRNA gene. PCR was accomplished using the Phusion® High Fidelity PCR Kit (New England Biolabs, Ipswich, Massachusetts, USA) according to the manufacturer's instructions. Then the target band was recovered by using QIAquick gel extraction kit (Qiagen, Hilden, Germany). The TruSeq® DNA PCR -Free Sample Preparation Kit (Illumina, San Diego, California, USA) was utilized to create libraries. Finally, the HiSeq2500 (Illumina, San Diego, California, USA) was used for sequencing in PE 250 bp mode.

**The 16S rRNA gene information analysis.** First, dual-terminal reads were merged with FLASH software (version 1.2.7). Next, QIIME 2 software (version 2020.11) was used for quality control to obtain clean tag and aligned the clean tag with the Gold database (version 20110519). Then used UCHIME software (version 7.0.1011) to detect the chimera sequence, and used Uparse software (version 7.0.1001), non-chimera clean tag were clustered into operational taxonomic units (OTU) according to the similarity threshold of ≥ 97%. Finally, mothur software was used to compare OTU with the SILVA database and for species taxonomic annotation. The MUSCLE software (version 3.8.31) was used to study the phylogenetic relationship of OTU with the SILVA database, and the number of OTU reads was normalized according to the minimum data size of the samples prior to analysis.

**NJ-associated gut microbiota analysis**. In order to screen the taxonomic components with significant differences between groups, the taxonomic components with low abundance (average relative abundance less than 0.1%) and low coverage (no taxonomic components were detected in more than 20% samples) were filtered out. The DESeq2 method was used to screen the taxonomy composition of gut microbiota with significant differences between groups, with parameters set at a *p* value of less than 0.05, a Benjamini and Hochberg false discovery rate (FDR) calibrated *p* value of less than 0.001, and| log2foldchange| >1.2.

**Similarity analysis.** Firstly, based on the genus level data, the vegdist function in R package Vegan is used to calculate the bray-curtis distance. Then, based on the obtained bray-curtis distance, the anosim function in R package Vegan is further used to calculate the sample similarity matrix. Finally, the stat_boxplot function in ggplot2 of R package is used for comparative analysis and visualization of similarities within and between groups.

**Partial least squares discriminant analysis (PLSDA).** In order to assess whether gut microbiome composition/metabolites that differed significantly between groups could distinguish NJ/HC, the plsda function of the R package mixOmics was alternatively used to perform PLSDA.

**3 Gut metabolome analysis**

**LC-MS/MS.** LC-MS/MS metabolomics detection is briefly described as follows: firstly, the metabolites were separated by liquid chromatography using Acquity Ultra Performance LC-QTOF system (Waters Corporation, Milford, MA, USA), then time-of-flight quantitative mass spectrometry (Waters Corporation) was used for further detection. The acquired data were processed by using Masslynx 4.1 software (Waters Company) to obtain a two-dimensional data matrix, which included retention time (RT), positive or negative mode mass-to-charge ratio (MZ), observed value (sample) and peak intensity.

**NJ-associated gut metabolite.** To screen for metabolite compositions that differed significantly between groups, metabolites with low abundance (average relative abundance < 0.001%) and low coverage (no metabolite detected in more than 20% of samples) were first filtered out. The DESeq2 method was used to screen for metabolites with significant differences between groups, with the parameters set to a *p* < 0.05, Benjamini and Hochberg false discovery rate (FDR) calibrated *p* < 0.001, and |log2foldchange| > 0.58. Filter_string = "0.5-0.01-0.05-0.05-0.05-0.05-0.58". Coverage Mean wilcox.p wilcox.fdr deseq2.p deseq2.fdr deseq2.logfoldchange.

**NJ-associated gut bile acids.** To further validate gut bile acids that differed significantly between groups, bile acids with low abundance (average relative abundance < 0.01%) and low coverage (no metabolite detected in more than 50% of samples) were first filtered out. The DESeq2 and Wilcoxon Rank Sum test methods were used to screen for metabolites with significant differences between groups, with the parameters set to a *p* < 0.05, Benjamini and Hochberg false discovery rate (FDR) calibrated *p* < 0.001, and with DESeq2|log2foldchange| > 0.58.

**Targeted metabolomic analysis.** Fecal bile acid detection was accomplished by the UPLC-QQQ-MS/MS method. A brief introduction is as follows. Firstly, sample preparation and extraction. The samples were thawed in a refrigerator at 4 ℃, and the appropriate weight of thawed samples was weighed and placed in a 2mL centrifuge tube, Add 600μl of ice-cold methanol, shake well for 1 min, grind for 5 min, let stand at 4 ℃ for 30 min, and centrifuge at 12000 rpm for 10 min. Then, a second addition of 600μl of ice methanol solution was added to the residue. The extracts were shaken well for 1 min, ground for 5 min, allowed to stand at 4℃ for 30 min, and centrifuged at 12000 rpm for 10 min. The two extracts were combined and blown dry. Finally, dissolved with 0.2ml methanol (containing 50ng/ml of internal standard), centrifuge for 15 min, extracted the supernatant. The supernatant was transferred to a clean test tube and dried in a rotary evaporation concentrator (Hunan Herexi Instrument Equipment Co., Ltd) and waited until it was tested on the machine. Secondly, UPLC-MS analysis. UPLC separation was performed in an Acquity Ultra Performance LC-QTOF system (Waters, UK) equipped with an Acquity UPLC BEH C18 (1.7 µm, 2.1 mm × 100 mm, Waters) column. The temperature of the column was set at 40℃. The sample injection volume was 3 µl. Eluents consisted in 0.05% formic acid in water (eluent A) and 0.05% formic acid in acetonitrile (eluent B). The flow rate was set at 0.3 ml/min. A 16-min elution gradient was performed as follows: during the first 1 min, eluent composition was set at 90% A and 10% B; then the proportion of B was increased to 40% in 2 min, followed by a further increase to 45% B reached at min 5. Further increase to 60% B reached at 7.5 min, and the proportion of B was increased to 65% in 9.5 min. Then further increase to 80% B reached at min 11.5 and kept for 2.5 min. Finally, the initial conditions were recovered and maintained for 2 min for column conditioning. The MS analysis was performed using a AB SCIEX 5500 QQQ -MS spectrometer (Waters) equipped with an ESI source in the positive/negative ion mode working in the multiple reaction monitoring (MRM) mode. A capillary voltage of 4.2kV, a source temperature of 450°C was used. Transitions, cone voltages, and collision energies were automatically tuned for each BA using the Quanoptimizer software (Waters). The data station operating software used was MassLynx 4.1 (Waters).

**4 Gut microbiome-metabolome association**

**Non-metric multidimensional scaling (NMDS).** Firstly, based on the genus level data, the metaMDS function in R package Vegan (version 2.6-4) is used for NMDS ranking analysis and the stress value is obtained. At the same time, adonis2 function in R package Vegan is used to carry out Permutative Multivariate Analysis of Variance (PERMANOVA) based on bray-curtis distance and get P value and R^2^ value. Then, the ordisurf function in R package Vegan is used to passively add environment variables to the NMDS ranking. Finally, the geom_point function in ggplot2 of R package is used to visualize the sorting results of NMDS.

**Constrained ordination analysis (Canonical Correlation Analysis/CCA, Redundancy analysis/ RDA).** Firstly, the data of genus level were selected as species composition, and the composition of metabolite markers is selected as environmental composition. The rda function in the R package Vegan (version 2.6-4) was used to perform RDA. Meanwhile, the cca function in the R package Vegan was used for CCA to obtained restrictive ranking relationships for species and environmental data. Then, the anova function in the R package Stats (version 4.2.3) was used to perform Permutation Multivariate Analysis of Variance (PERMANOVA), calculating *p* values and R-squared values (R^2^) for restrictive rankings such as RDA and CCA. Finally, after taking the first two axes and removing the largest deviations, the restrictive ranking relationships were visualized and displayed through the geom_point function in ggplot2 of R package.

**Survival analysis.** Kaplan-Meier survival curve is completed by KaplanMeierFitter function of Lifelines package (version 0.26.4) of Python software (version Python 3.7.6). When conducting univariate survival analysis, we first set up the time variable and the event variable and compared the differences in univariate survival analysis. Statistical significance is achieved by the logrank_test function of the statistics module of the lifelines package, and *p* value of less than 0.05 is regarded as significant. Multivariate survival analysis was done through the lifelines package CoxPHFittert function.

**5 Causal inference analysis**

**Causal mediation analysis.** First, two statistical models are developed, namely the model fitY (fitY = lm(Y ~ X + M)) for the dependent variable (Y) on the independent variable (X) and the model fitM (fitM = lm(M ~ X)) for the mediator variable (M) on the independent variable (X). The gvlma function of the R package gvlma is used to fit these two models, then the R package mediation (version 4.5.0) of the mediate function was used to perform mediation analysis with the parameters set to boot=TRUE, sims=999, which obtained the indirect causal effects of the independent variable (X) on the dependent variable (Y) through the mediator variable (M) by being able to call a bootstrap with a sampling number of 999 (average causal mediation effects, ACME) size and p-value, and to obtain the size and p-value of the direct effects (average direct effects, ADE) of the independent variable (X) directly on the dependent variable (Y). Finally, the R packageggalluvial was used to visualize the analysis of causal mediation effects.

**Machine learning-causal inference.** A brief description is as follows: the first step is to set up a modeling, that is, to encode our domain knowledge into a causal model and represent it with a graph, each arrow in the graph represents a causal relationship: "A->B" means that the variable A leads to the variable B. We set up the outcome as a binary variable, where 0 represents the control group and 1 represents the disease group. group name binary variable, where 0 represents the control group, 1 represents the disease group, set the intervention (treatment) of the variable as a potentially important clinical indicators leading to disease, and other variables to be examined may be the common cause of the intervention (treatment) and the outcome as confounders. In the second step, identification, which is based on Dowhy's backdoor.linear_regression method, is performed to check whether the target quantities can be estimated given the observed variables. The third step, estimation, is to construct an estimator to compute the estimand identified in the previous step. To model nonlinear data (as well as data with high-dimensional confounders), we constructed the estimator using EconML's machine learning method that uses gradient boosting trees to learn the relationship between the outcome and the confounders, as well as the relationship between the intervention and the confounders, and finally compares the residuals between the outcome and the intervention.

**6 Clinical predictive modeling**

**Accuracy assessment.** The calibration curve is an important indicator to evaluate the accuracy of a disease risk model in predicting the probability of an outcome event occurring in an individual in the future; a good calibration suggests that the prediction model is highly accurate, and a poor calibration suggests that the model is likely to overestimate or underestimate the risk of disease occurrence. We plotted the calibration curve (Calibration) of the clinical prediction model by R package riskRegression (version 2020.12.08), firstly, we fit the model by glm function, then, we calculated the auc and Brier scores of the model by Score function, and finally, we calculated the auc and Brier scores of the model by plotCalibration function to visualize the calibration curve.

**Clinical effect assessment.** Traditional diagnostic test metrics such as sensitivity, specificity and area under the ROC curve measure only the diagnostic accuracy of a predictive model and fail to take into account the clinical utility of a particular model, Decision Curve Analysis (DCA) has the advantage that it integrates the patient benefit into the clinical analysis. We plotted the clinical decision curves through the plot_decision_curve function of the R package rmda, and we also plotted the clinical impact curves through the plot_clinical_impact function to assess the clinical utility of the models.

**Risk prediction.** The probability of occurrence of a clinical outcome or a certain type of event is realized by means of a nomogram, which is based on the principle of constructing a multifactorial regression model, scoring the level of each value according to the degree of influence of each influencing factor on the outcome variable in the model, i.e., according to the magnitude of the regression coefficients, and then summing the scores to obtain a total score, and finally calculating the predicted probability of occurrence of a single outcome event by means of the functional transformation of the total score to the probability of occurrence of the outcome event. predicted probability of the outcome event. We first fit different logistic regression models through the lrm function of the R package rms, and then plotted the column plots through the regplot function of the R package regplot.

**7 Machine learning models**

**Lasso model prediction.** Lasso regression analysis was performed using the lasso function of the scikit-learn linear_model module. First, the parameters were tuned using the GridSearchCV function of the scikit-learn model_selection to find the optimal alpha parameter of the lasso regression model; alpha was set as [1e − 5, 1e − 4, 1e − 3, 1e − 2, 1, 5, 10, 20]. Then, the lasso regression model, constructed with the optimal parameters, was used to fit the training dataset. Finally, the fitted model was used to predict the target value of the test dataset, and the top 10 lasso regression coefficients were used for data visualization, which was done using the barh function of the matplotlib library (version 3.2.2).

**Lasso + XGBoost model prediction.** Because the performances of lasso and XGBoost were both good, we combined the two models for the regression analysis. Considering that lasso performed better than XGBoost on the training dataset, we set the weight of lasso as 0.6 and the weight of XGBoost lower as 0.4. The prediction value of the test dataset was (predictions_test) = np.expm1(0.6 × lasso_pred_test + 0.4 × y_pred_xgb_test), where np.expm1 is the inverse operation of the log1p function, lasso_pred_test is the lasso prediction value, and y_pred_xgost_test is the XGBoost prediction value.

**Random forest machine learning evaluation.** First, used the train_test_split function in the model_selection module of the sklearn package (version 0.23.1) in Python (version 3.7.6) and split all samples into training and test datasets, set parameters: test_size=0.4, random_state =2020. Import the RandomForestClassifier from the collection module of the sklearn package as a RandomForest model And set the parameters of RandomForest model as: random_state=0, n_estimators=100, oob_score=True, n_jobs=-1. The RandomForest model is fitted according to the training data set. Used the feature_importances_ function of the RandomForest model to rank the importance of the features and used the barh function from the pyplot module of the matplotlib package in Python (version 3. 2.2) for visualizing important features. Finally, Roc_curve and auc functions in the metrics module of sklearn package are used to analyze the ROC and evaluate the AUC score of the test samples respectively, and used plot module of matplotlib package for visualization. In order to get the 95% confidence interval of the AUC scores on the test samples, Use the cross_val_score function of model_selection module in sklearn package for cross validation analysis, with a parameter of cv = 3, and the 95% confidence intervals were computed as: scores_cv.mean ± scores_cv.std * 1.96.

**8 Supplementary Tables**

**Clinical characteristic of study population**

**Table 1 discovery stage**

|  | HC (n=68) | NJ (n=68) | *P* |
| --- | --- | --- | --- |
| Male sex, N (%) | 35 (51.5) | 38 (55.9) | 0.731 |
| Preterm, N (%) | 16 (23.5) | 4 (5.9) | 0.008 |
| Vaginal delivery, N (%) | 44 (64.7) | 55 ( 80.9) | 0.054 |
| Antibiotic use, N (%) | 47 (69.1) | 0 (0.0) | <0.001 |

**Table 2 validation stage**

|  | HC (n=30) | NJ (n=30) | *P* |
| --- | --- | --- | --- |
| Male sex, N (%) | 11 (36.7) | 18 (60.0) | 0.071 |
| Gestationa age, weeks | 38.90 (1.95) | 38.60 (1.19) | 0.476 |
| Vaginal, N (%) | 10 (33.3) | 27 (90.0) | <0.001 |
| Antibiotics use, N (%) | 15 (50.0) | 2 (6.7) | 0.001 |

1. **Supplementary figures**

**
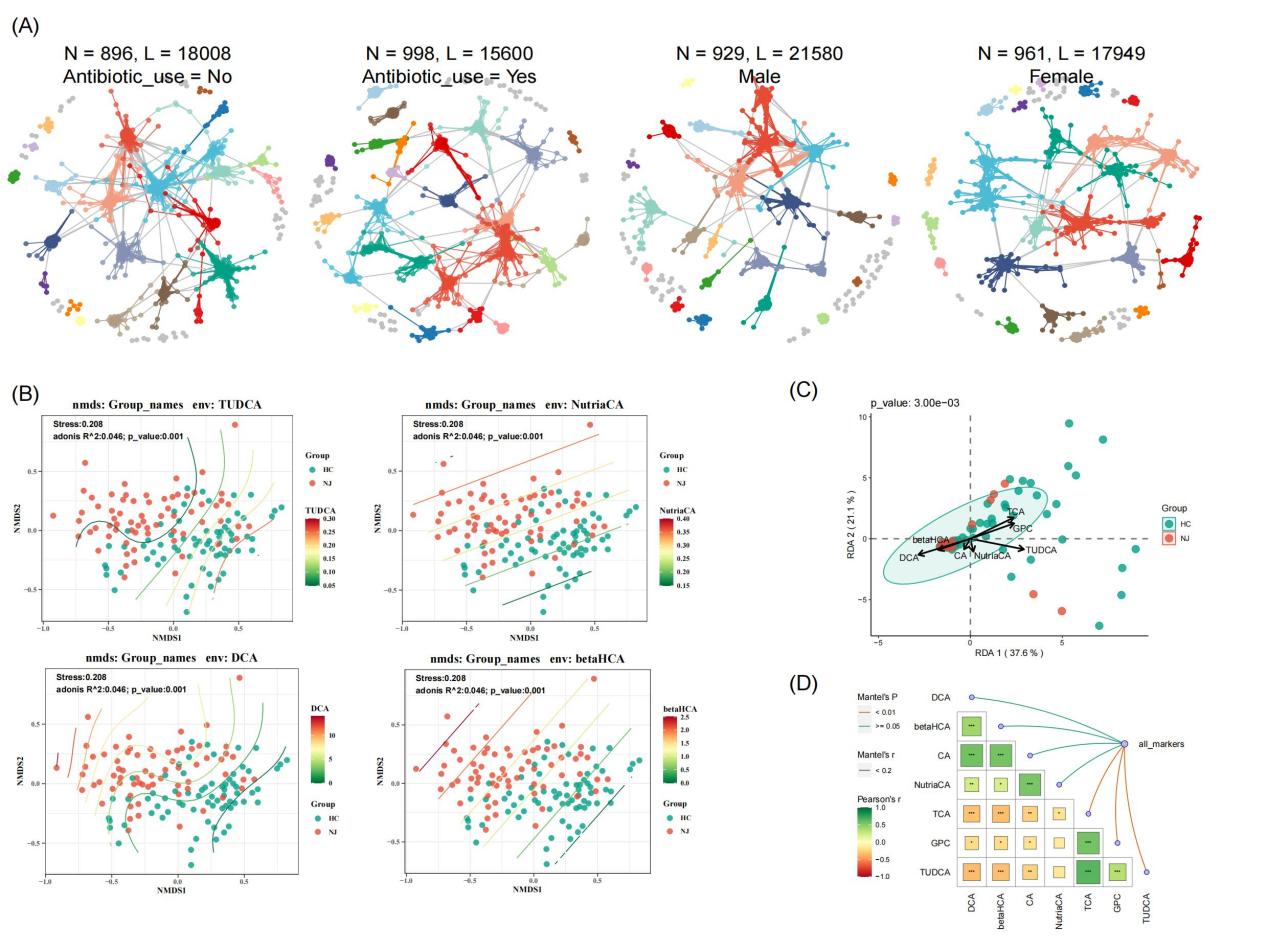
**

**Figure S1. NJ-associated gut bacteria network module**. (A) The 16S rRNA sequencing-based MENs methods and visualization tools reveal the interrelationships among gut microbes between groups; (B) NMDS analysis showed significant clustering of intestinal bile acids TUDCA, NutirCA, DCA and betaHCA between the NJ and HC groups; (C) RDA analysis found that there was a correlation between gut microbiota and bile acid markers; (D) Mantel analysis showed a strong correlation between gut bacterial markers and bile acids.


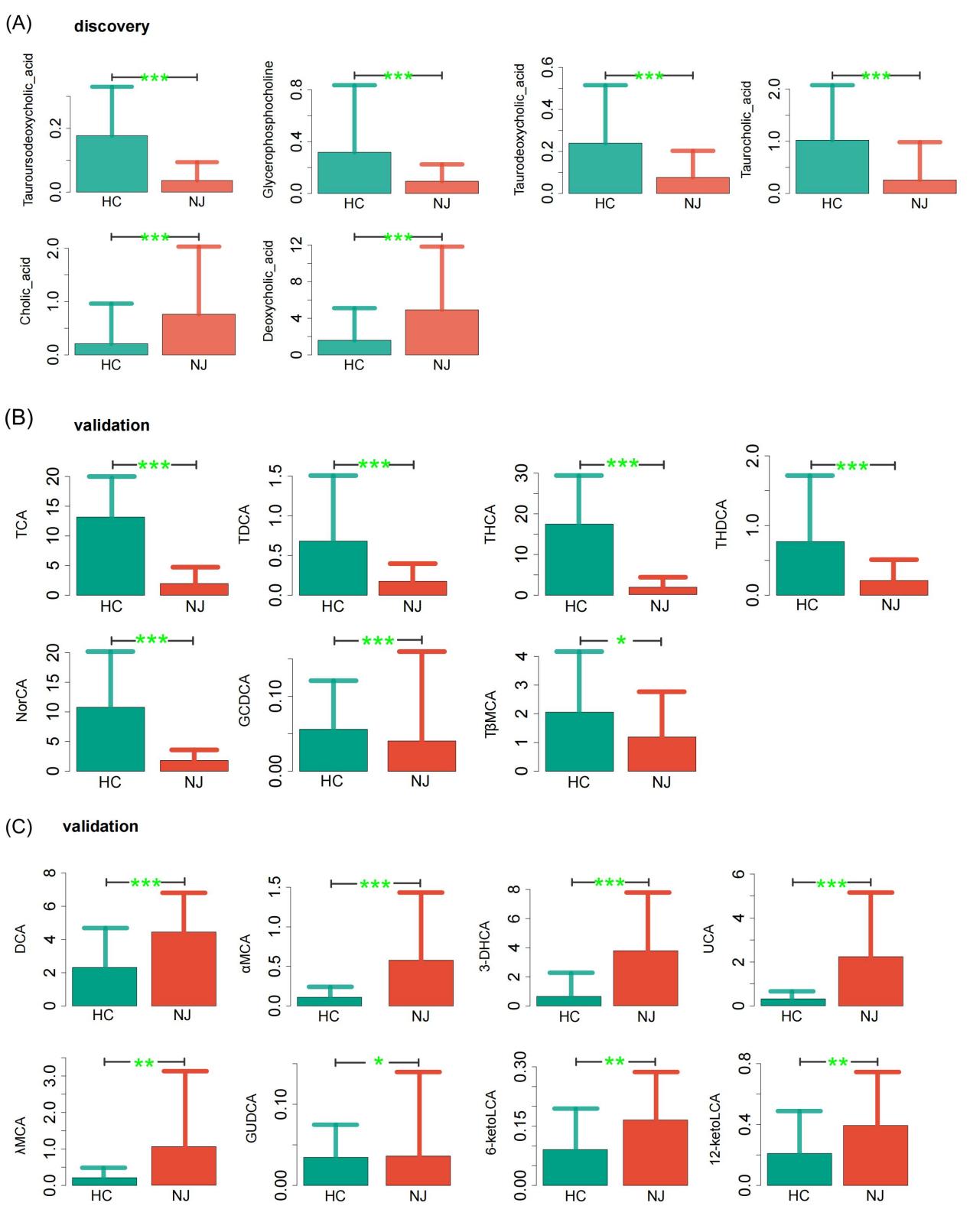


**Figure S2. Significant difference in bile acid levels between NJ and HC groups.** (A) In discovery stage, the levels of bile acids TUDCA, TDCA, TCA and bile acid derivative glycerophosphocholine were significantly higher in the HC group than in the NJ group, the levels of bile acids DCA and CA were significantly higher in the NJ group than in the HC group; (B-C) In validation stage, the levels of bile acids TCA, TDCA, THCA, THDCA, NorCA, GCDCA, TβMCA and bile acid derivative glycerophosphocholine were significantly higher in the HC group than in the NJ group, the levels of bile acids DCA, αMCA, 3-DHCA, UCA, λMCA, GUDCA, 6-ketoLCA and 12-ketoLCA were significantly higher in the NJ group than in the HC group (*** *P* < 0.001, ** *P* < 0.01, * *P* < 0.05).

**
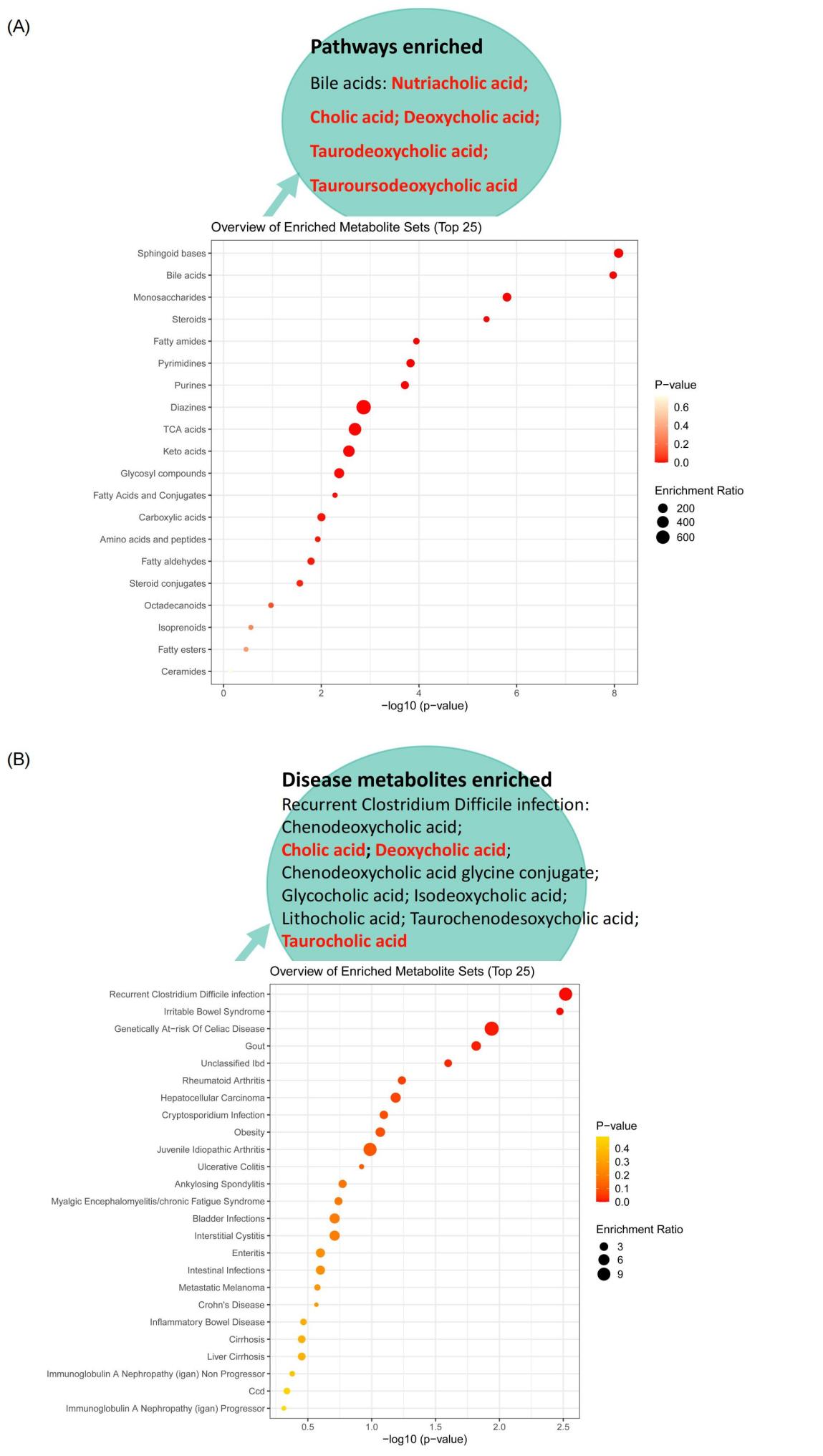
**

**Figure S3. NJ-related metabolite enrichment analysis.** (A) Biological pathway enrichment analysis was performed for the differential metabolites, and significant enrichment was found for the bile acid-related pathway; (B) Disease enrichment analysis was performed for the differential metabolites, and significant enrichment was found for *Recurrent Clostridium* difficile infection in which the enriched metabolites were bile acid metabolites.


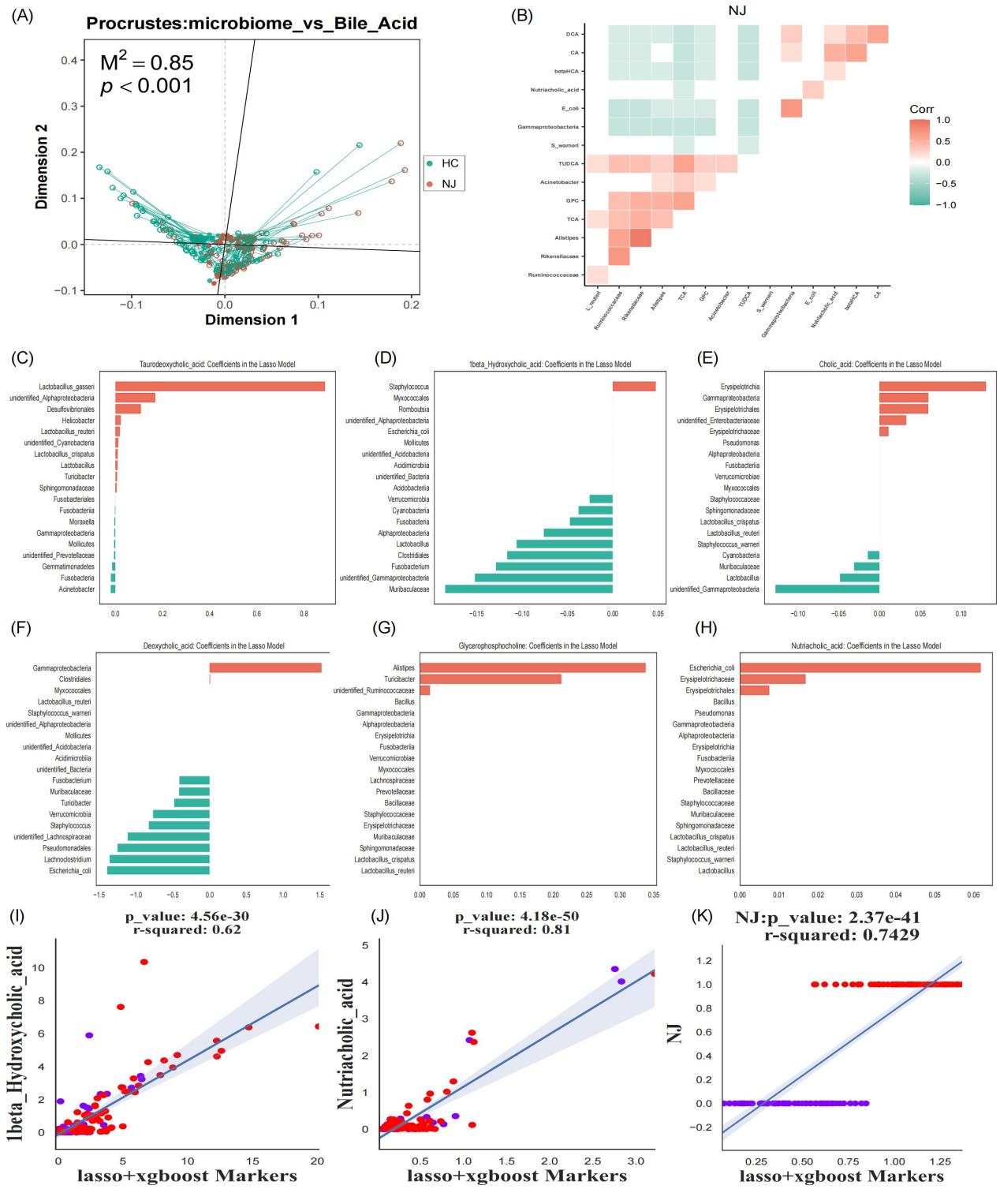


**Figure S4. Machine learning approach finds gut bacteria can predict bile acid levels and NJ.** (A) A strong correlation between NJ-associated gut microbiota composition and bile acids was found by Procrustes analysis; (B) Correlation heatmap reveals correlation between NJ-associated gut bacteria and bile acids; (C) NJ-associated gut bacteria are significantly correlated with bile acids; (D) Gut bacteria affecting gut Taurodeoxycholic acid are *L. reuteri* in positive direction; (E) Gut bacteria affecting gut 1beta Hydroxycholic acid are *Rikenellaceae* and *Ruminococcaceae*, with a negative direction; (F) The gut bacteria affecting gut Cholic acid are *Gammaproteobacteria* and *Erysipelotrichales* with a positive direction, while *Ruminococcaceae* have a negative effect on Cholic acid; (G) Gut bacteria affecting gut Deoxycholic acid were *Gammaproteobacteria* with a positive direction, while *Rikenellaceae* and *Ruminococcaceae* also had an effect on Deoxycholic acid with a negative direction; (H) Gut bacteria affecting gut Glycerophosphocholine were *Alistipes* and *Acidobacteria* with positive direction; (I-J) Based on gut bacteria abundance can predict gut bile acid 1beta Hydroxycholic acid (I) and Nutriacholic acid (J) content; (K) Prediction of NJ based on gut bacteria abundance (considering NJ as 1 and HC as 0).


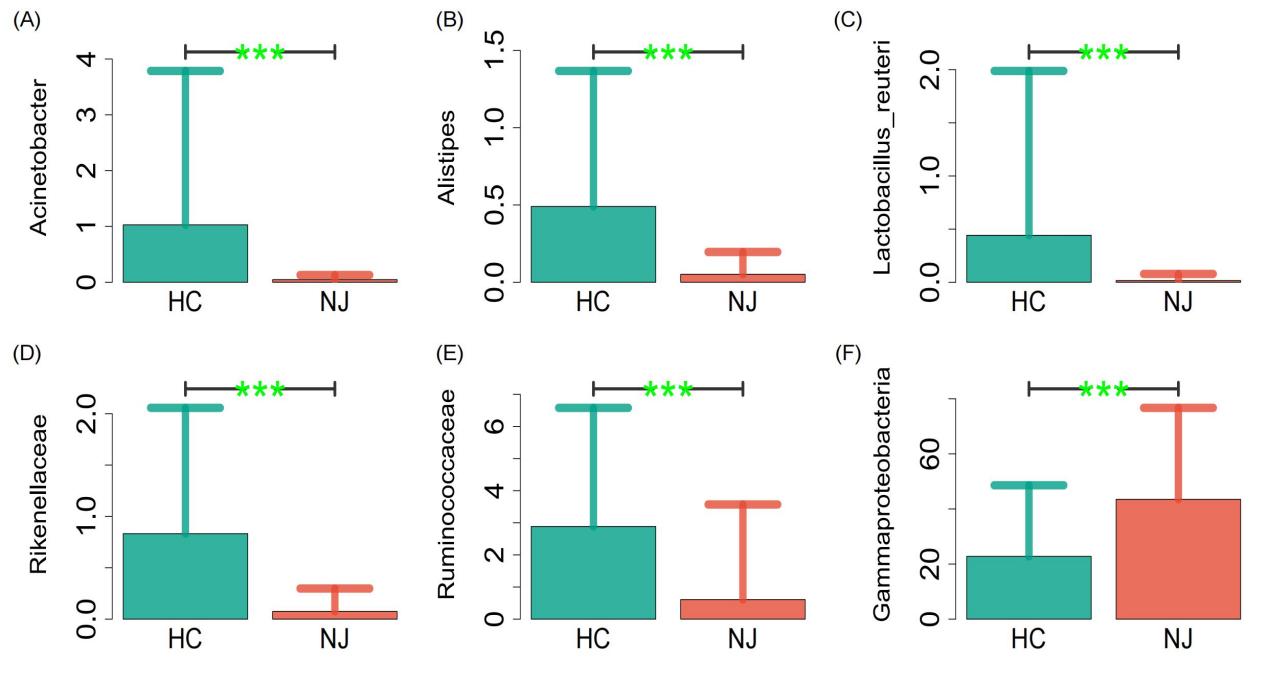


**Figure S5. Focused NJ-associated gut bacteria.** (A-E) Five gut bacteria *Acinetobacter* (A), *Alistipes* (B), *L. reuteri* (C), *Rikenellaceae* (D), *Ruminococcaceae* (E) that significantly decreased in the NJ group compared to the HC group; (F) Gut bacteria *Gammaproteobacteria* that significantly increased in the NJ group compared to the HC group.


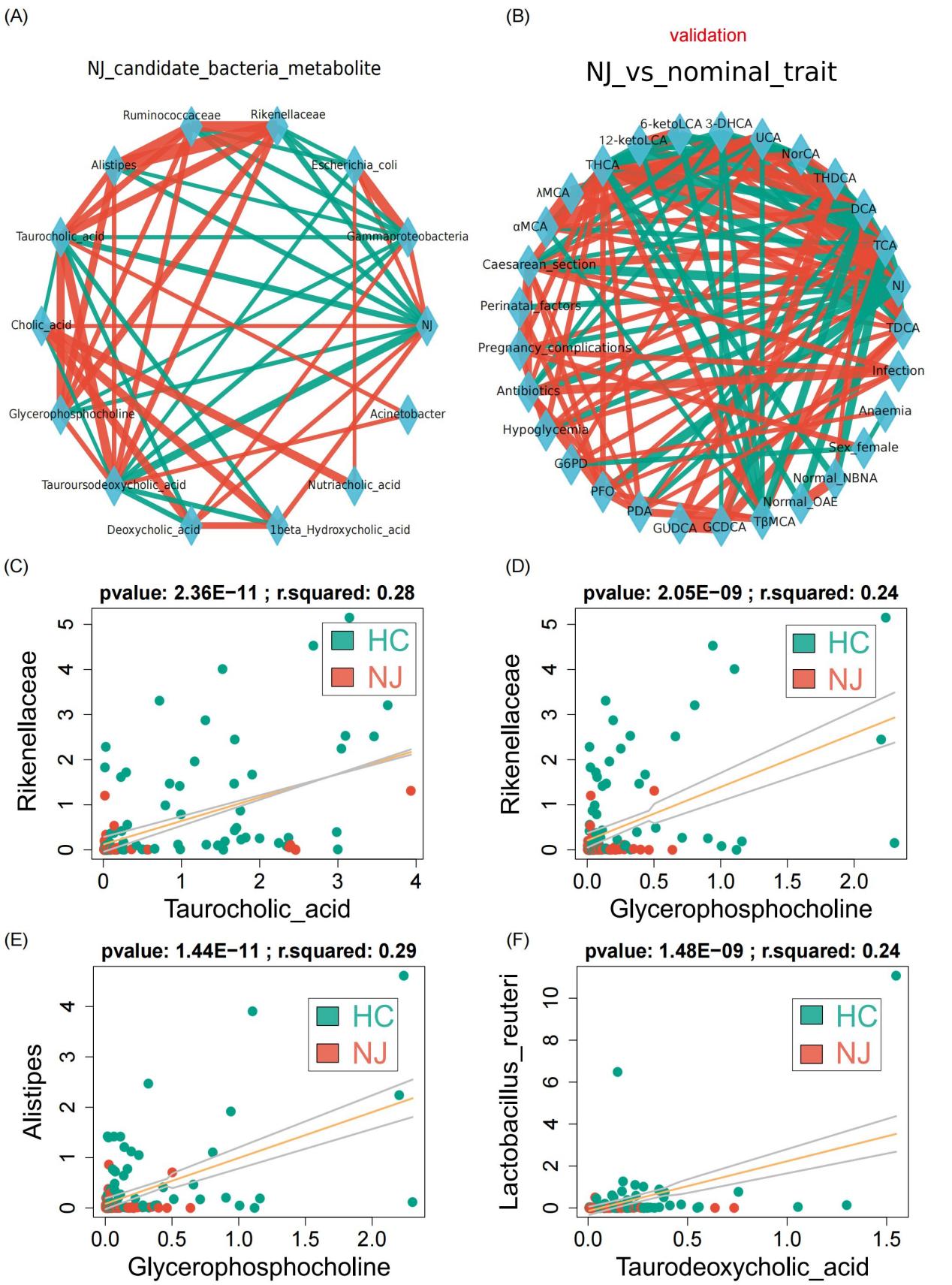


**Figure S6. Network and linear relationship between NJ-related gut bacteria and bile acids.** (A-B) Complex network relationships between NJ-associated gut bacteria and bile acids in the discovery (A) and validation (B) phases; (C) Gut abundance of gut bacteria *Rikenellaceae* positively correlated with gut bile acid TCA content; (D) Gut abundance of gut bacteria *Rikenellaceae* positively correlated with gut bile acid derivative Glycerophosphocholine; (E) Positive correlation between the gut abundance of the gut bacterium *Alistipes* and the gut bile acid derivative Glycerophosphocholine; (F) Positive correlation between the abundance of the gut bacterium *L. reuteri* and the gut bile acid TDCA.


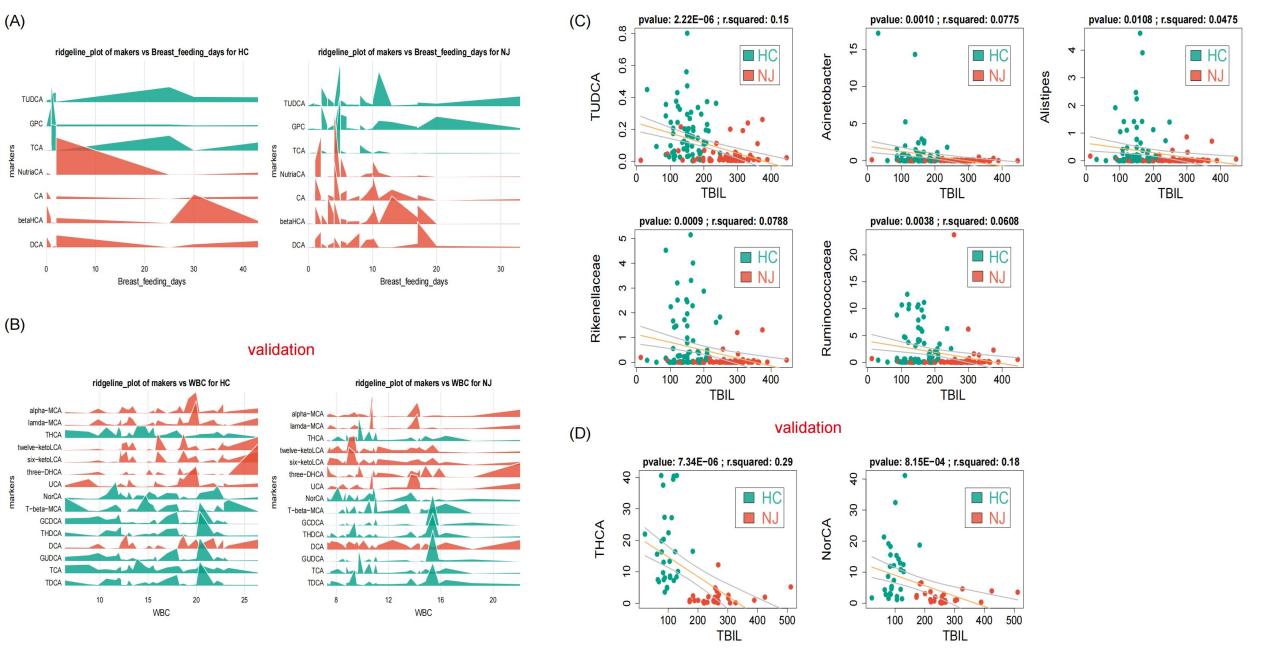


**Figure S7. Correlation between NJ-related intestinal bacteria and bile acids and clinical phenotype.** (A-B) [Ridgeline plot](http://www.baidu.com/link?url=Lq_w5DiXkaOnRXR2DVkhzPGxbo_nmDxOzpOOUA1XUh-kp4tqdmauhKo5shSanSIdfptr6MeDw8fz8yDDNz6KjCgyBJuFZIslpcp9W17qOJK) showed the distribution of bile acids in the NJ and HC groups at different breastfeeding durations and different leukocyte levels; (C) Secondary bile acids TUDCA were negatively correlated with serum total bilirubin levels, and gut bacteria *Acinetobacter*, *Alistipes*, *Rikenellaceae*, and *Ruminococcaceae* affecting bile acid content were negatively correlated with serum total bilirubin levels; (D) Bile acids THCA and NorCA were negatively correlated with serum total bilirubin levels in the validation stage.


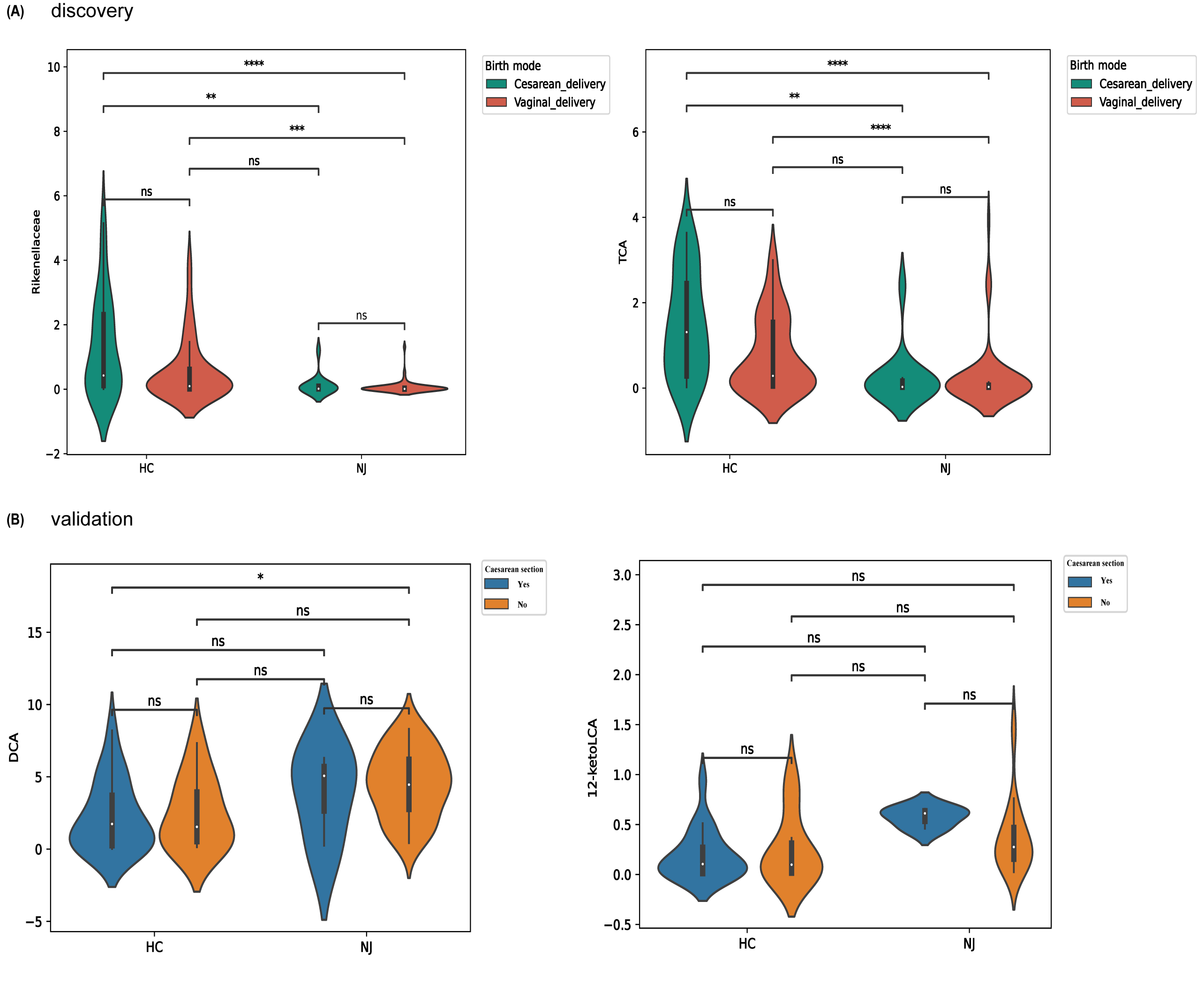
**Figure S8. Correlation analysis of the proportion of gut microbiota and bile acid marker composition with clinical phenotypes in each group.** The vertical coordinate represents the bacterial/bile acid composition ratio; larger values indicated higher proportions in the group where the organism/bile acid was located. (A) There was a significant difference between *Rikenellaceae* and TCA for different modes of delivery between the two groups in the discovery stage; (B) There were no difference between DCA and 12-ketoLCA between the two groups with different modes of delivery in the validation stage. The ns in the figure means no statistical difference. * *P* < 0.5, ***P* < 0.01, *** *P* < 0.001.


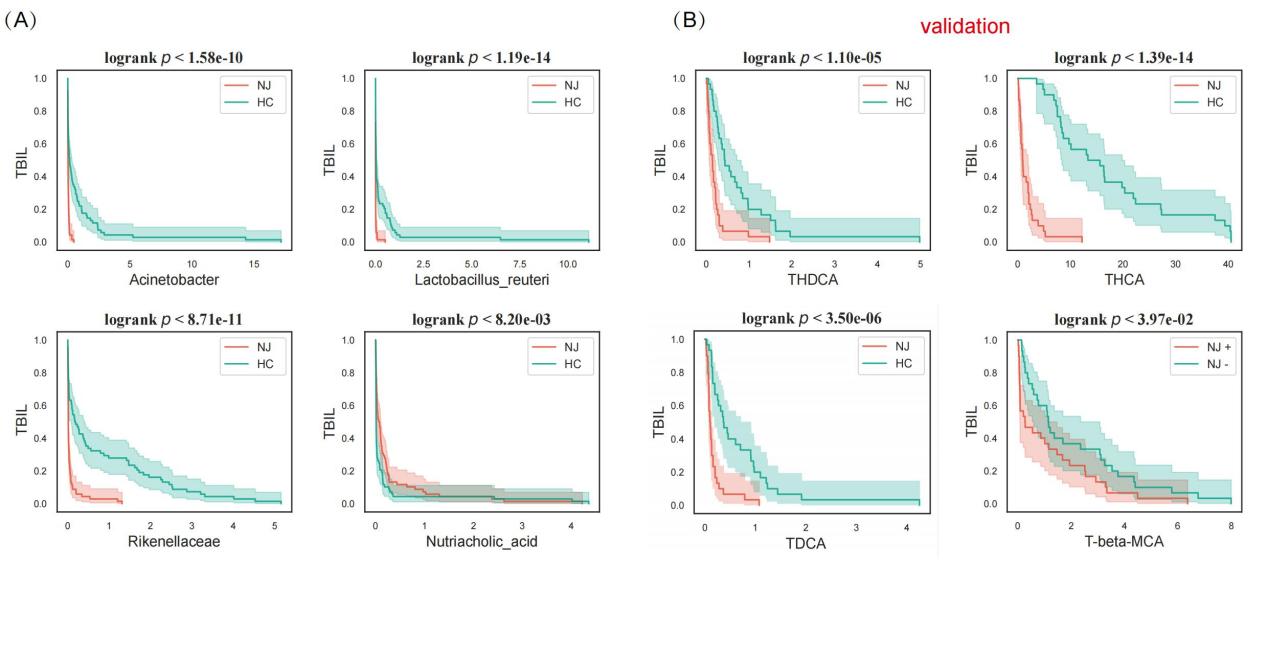
**Figure S9. Survival analysis to assess the relationship between NJ-associated gut bacteria and bile acid metabolites and TBIL.** (A) In the discovery stage, as the abundance of gut *Acinetobacter*, *L. reuteri* bacteria decreased, the magnitude of the rise in TBIL was significantly different between the NJ and HC groups; with the rise in gut primary bile acids CA and bile acid derivative Nutriacholic acid, the difference in the rise in TBIL between the NJ and HC groups was significant, with a larger rise in the NJ group; (B) In the vaidation stage, with the decline in gut bile acids THDCA, THCA, TDCA and TβMCA, the difference in the magnitude of the rise in TBIL between the NJ and HC groups is significant, with a greater rise in the NJ group.


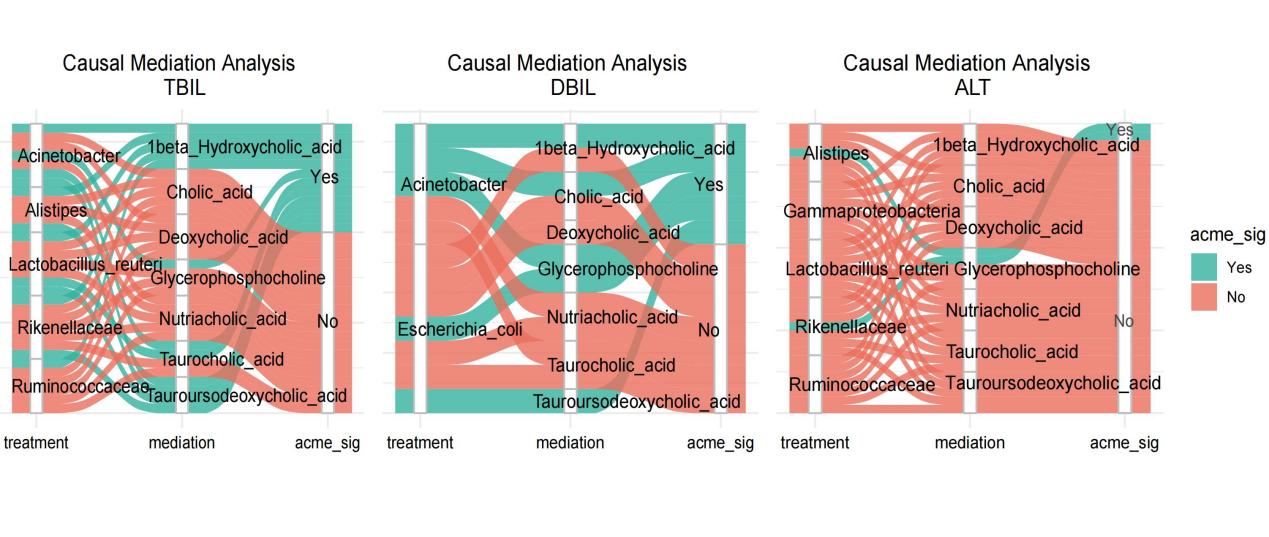


**Figure S10. Causal mediation analysis to assess the potential causal effects of bile acid-related bacteria, bile acid with NJ and clinical indicators.** The alluvial diagram visualizes the results of causal mediation analysis. For each subplot, the three columns on the horizontal axis indicate the causal effect of the intervention variable (treatment), the mediating variable (mediation) and the intervention outcome, respectively. Causal mediation analysis showing that gut bacteria affect TBIL, DBIL and ALT by influencing bile acid levels.


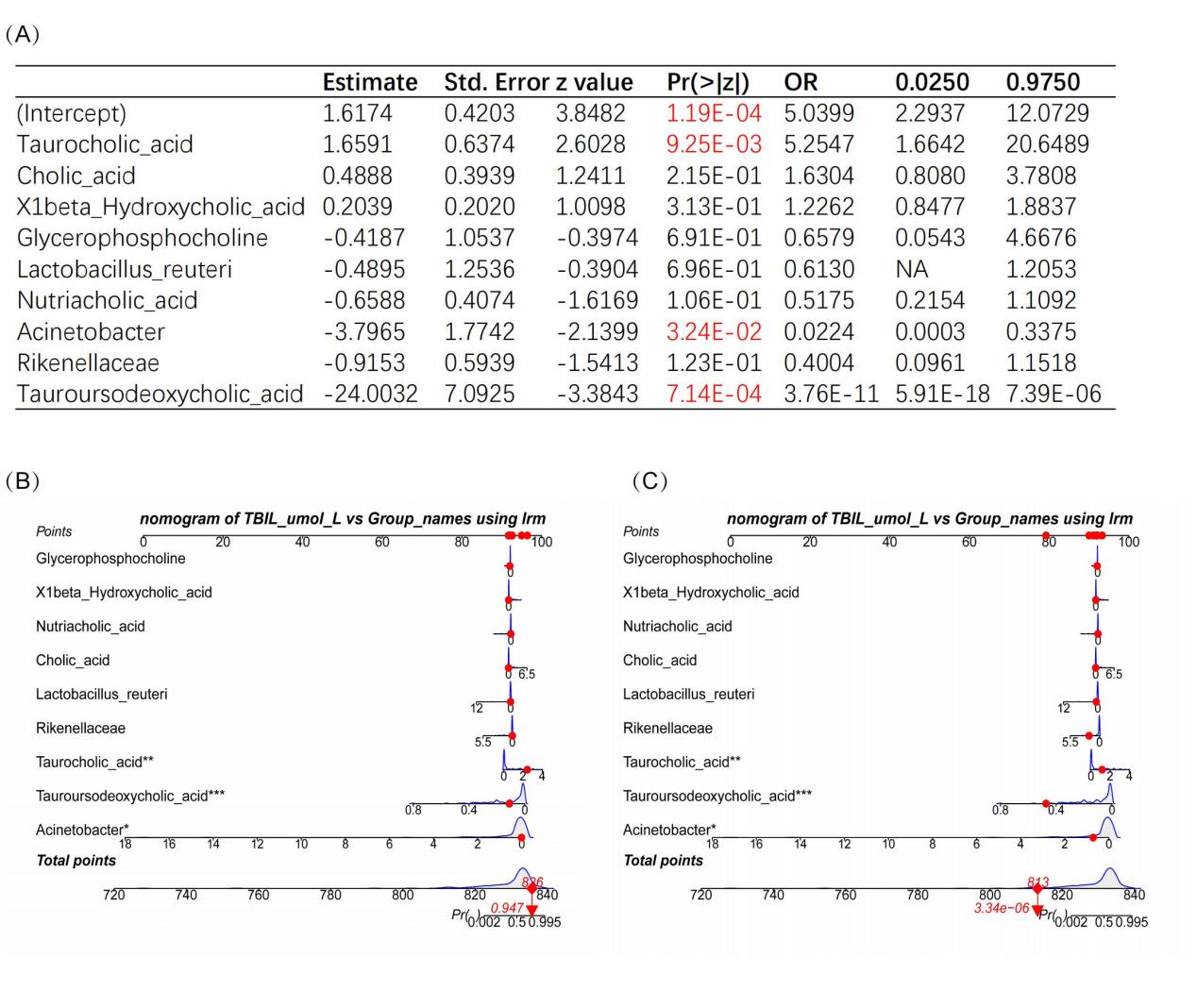


**Figure S11. Risk prediction for jaundice disease based on 9 clinical variables.** (A) The optimal Lasso model evaluation results, it is seen that TCA, *Acinetobacter*, and TUDCA have the most significant effects on the model; (B) The corresponding scores of each clinical indicator for a patient with NJ, the total score situation, the risk for this patient is 0.947; (C) The corresponding scores of each clinical indicator for an individual with HC, the total score situation, the risk for this individual is close to 0.
